# Transcriptomics profiling of the non-small cell lung cancer microenvironment across disease stages reveals dual immune cell-type behaviors

**DOI:** 10.3389/fimmu.2024.1394965

**Published:** 2024-10-31

**Authors:** Marcelo Hurtado, Leila Khajavi, Abdelmounim Essabbar, Michael Kammer, Ting Xie, Alexis Coullomb, Anne Pradines, Anne Casanova, Anna Kruczynski, Sandrine Gouin, Estelle Clermont, Léa Boutillet, Maria Fernanda Senosain, Yong Zou, Shillin Zhao, Prosper Burq, Abderrahim Mahfoudi, Jerome Besse, Pierre Launay, Alexandre Passioukov, Eric Chetaille, Gilles Favre, Fabien Maldonado, Francisco Cruzalegui, Olivier Delfour, Julien Mazières, Vera Pancaldi

**Affiliations:** ^1^ CRCT, Université de Toulouse, Institut national de la santé et de la recherche médicale (Inserm), Centre national de la recherche scientifique (CNRS), Université Toulouse III-Paul Sabatier, Centre de Recherches en cancérologie de Toulouse, Toulouse, France; ^2^ Department of Medicine, Vanderbilt University Medical Center, Nashville, TN, United States; ^3^ Laboratory Medicine, Oncopole Claudius Regaud, Toulouse, France; ^4^ Institut de Recherche Pierre Fabre, Toulouse, France; ^5^ Pulmonology Department, Larrey Hospital, University Hospital of Toulouse, Toulouse, France; ^6^ Division of Allergy, Pulmonary, and Critical Care Medicine, Department of Medicine, Vanderbilt University Medical Center, Nashville, TN, United States; ^7^ Cancer Early Detection and Prevention Initiative, Vanderbilt-Ingram Cancer Center, Vanderbilt University Medical. Center, Nashville, TN, United States; ^8^ Department of Biostatistics, Vanderbilt University Medical Center, Nashville, TN, United States; ^9^ Data Science, Centre Hospitalier Universitaire de Toulouse, Toulouse, France; ^10^ Life Sciences Department, Barcelona Supercomputing Center, Barcelona, Spain

**Keywords:** lung adenocarcinoma, natural killer cells, immune landscape, cell deconvolution, transcription factor activity

## Abstract

**Background:**

Lung cancer is the leading cause of cancer death worldwide, with poor survival despite recent therapeutic advances. A better understanding of the complexity of the tumor microenvironment is needed to improve patients’ outcome.

**Methods:**

We applied a computational immunology approach (involving immune cell proportion estimation by deconvolution, transcription factor activity inference, pathways and immune scores estimations) in order to characterize bulk transcriptomics of 62 primary lung adenocarcinoma (LUAD) samples from patients across disease stages. Focusing specifically on early stage samples, we validated our findings using an independent LUAD cohort with 70 bulk RNAseq and 15 scRNAseq datasets and on TCGA datasets.

**Results:**

Through our methodology and feature integration pipeline, we identified groups of immune cells related to disease stage as well as potential immune response or evasion and survival. More specifically, we reported a duality in the behavior of immune cells, notably natural killer (NK) cells, which was shown to be associated with survival and could be relevant for immune response or evasion. These distinct NK cell populations were further characterized using scRNAseq data, showing potential differences in their cytotoxic activity.

**Conclusion:**

The dual profile of several immune cells, most notably T-cell populations, have been discussed in the context of diseases such as cancer. Here, we report the duality of NK cells which should be taken into account in conjunction with other immune cell populations and behaviors in predicting prognosis, immune response or evasion.

## Background

Lung adenocarcinoma exhibits diverse clinical behaviors, ranging from indolent to aggressive metastatic disease. However, the biological underpinnings of this heterogeneity remain poorly understood. Non Small Cell Lung Cancer (NSCLC) is often diagnosed at an advanced stage and its management is currently undergoing significant transformation. Molecular testing, targeted therapies, and immunotherapy are now part of routine clinical care (1). However, despite major progress in the therapeutic management of NSCLC cancer, many patients are still refractory to the initial treatment or develop resistance leading to tumor recurrence. Furthermore, the clinical and pathological diversity of NSCLC is associated with a highly complex genomic landscape and heterogenous immune tumor microenvironment. Interactions between tumor cells and the immune microenvironment are known to profoundly impact cancer pathogenesis and progression (2).

Lung cancer tumor biopsies contain a heterogeneous mix of cancer cells, healthy cells, immune cells, and extracellular factors that constitute the tumor microenvironment (TME). The specific composition and functional profiles of immune cells within the TME can profoundly influence tumor pathogenesis. Detailed characterization of immune cell diversity in the TME has therefore become a major goal in cancer research. However, dissecting the immune landscape from bulk tumor profiling remains challenging (3–5). Single cell RNA sequencing enables high-resolution dissection of tumor-immune interactions, but remains prohibitively costly for large-scale or clinical applications (6). Additionally, each single cell isolation approach introduces distinct technical biases that can skew rare cell detection. Computational deconvolution approaches can leverage unique gene expression signatures to estimate immune cell subsets from bulk transcriptomics in a more accessible and standardized way (7). However, numerous deconvolution algorithms exist with little consensus on best practices. In this study, we performed an integrated analysis using bulk RNAseq and validating our results with single cell RNA sequencing data. We applied this multi-omics pipeline to understand heterogeneity specifically in the microenvironment of early-stage lung adenocarcinomas, for which could validate our results on an independent cohort and on early stage lung adenocarcinoma (LUAD) samples from TCGA. We further correlated immune deconvolution features with clinical outcomes, highlighting the potential value of our approaches to reveal clinically relevant cellular populations and potentially implicating distinct NK cell phenotypes in survival. By correlating the deconvolution immune cell estimates and inferred transcription factor activities, we aimed to overcome limitations of individual methods. This study provides a framework for robust characterization of tumor immune landscapes from bulk transcriptomics.

## Methods

### Patient summary

The primary analysis cohort was derived from a pilot study stemming from a collaborative effort between l’Institut Universitaire du Cancer de Toulouse (IUCT) and Institut de Recherche Pierre Fabre (IRPF) aimed at assessing the technical feasibility of developing molecular characterization of lung tumors in order to enrich the activities already initiated by the IUCT. Patients were enrolled in the study if they were diagnosed with non-small cell lung cancer (NSCLC). Patients were excluded from this study if they were treated for any NSCLC prior to study enrollment. All individuals involved signed a non-objection form to part-take in the research program under the LUNG PREDICT protocol. Blood samples were gathered as part of a collection declared to the Ministry of Research under the number DC-2011-1382. Tissue samples are the remaining parts of the whole tissue belonging to the patient coming from the tumor library of CHU Biological Resource Center (IUCT-O) declared to the Ministry of Research under the number DC-2008-463. All clinical, pathological and molecular data were prospectively collected. Patients’ therapeutics and outcome were collected overtime with a 33 months median follow-up.

### Sample selection and extraction

A certified pathologist made the selection of slides with haematoxylin eosin slide coloration. The paraffin embedded block was cut, 1 HE to control the extraction and 4 to 16 sections of 10 *µ*m for the RNA extraction, which was performed with High Pure FFPE RNA extraction kit from Roche (Ref 0665077500). The purified RNA samples were analyzed with Fragment Analyser (Advanced Analytical Technologies Inc., Agilent Technologies, US) and High Sensitivity RNA Kit (DNF-472-0500, Agilent Technologies, US) to determine the RIN and the DV200 (percentage of RNA *≥* 200 bp).

### RNA sequencing

The libraries were prepared with the KAPA RNA HyperPrep Kit with RiboErase (HMR) (Kapa/Roche KK8560) for whole transcriptome sequencing as recommended by the supplier using 1 *µ*g input of RNA. Briefly, rRNA was hybridized with DNA probes to 5S, 8.8S, 18S, 28S, 12S and 16S rRNA, then the hybrids were depleted by enzymatic depletion using RNAse H. After, DNA digestion and fragmentation with high temperature were done. First strand, second strand synthesis and A-tailing were performed. Next, adapters from 1.5-7 *µ*M depending on the DV200 were ligated and the library was amplified. Library size and quality were confirmed on Fragment Analyzer (Advanced Analytical Technologies Inc., Agilent Technologies, US) and High Sensitivity NGS Fragment Analysis Kit (DNF-474-0500, Agilent Technologies, US). Qubit (ThermoFisher Scientific, US) was used to quantify libraries. Samples were pooled in equimolar fashion (10nM), then denatured and 1.8 pM was sequenced on NextSeq 550 (Illumina, US) in pair-end sequencing (76 bp reads) and double index 8 bp with NextSeq 500/550 High Output kit v2.5, 150 cycles (20024907, Illumina, US) and 1% PhiX (FC-110-3001, Illumina, US).

### Bulk RNAseq sample processing

Raw sequences were quality checked using FastQC (8 (v0.11.2)) and FastqScreen (9 (v0.15.2)) prior to aligning to the Homo sapiens primary genome sequence (Gencode: GRCh38, v27) using STAR (10 (v2.7.10a)) with encode options. FastQC was again used to assess the mapping quality. RSEM (11 (v1.3.1)) was used to generate the expression matrix (featureCounts from Rsubread R package (12 (v1.22.2)) was used for validation data).

### Differential expression analysis

Expression matrices from bulk RNAseq were analyzed with DESeq2 (13 (v1.42.1)) in the R environment (14); R Core Team (15) (version v4.2.3, BioConductor version v3.9 (16, 17) to identify differentially expressed genes (DEGs) between samples groups. ClusterProfiler (18 (v4.4.4)) was used to classify the DEGs into KEGG pathways. Heatmaps were generated using both pheatmap (v1.0.12) and ComplexHeatmap (19 (v2.0.0)) R packages. Volcano plots were generated using the EnhancedVolcano (20 (v1.2.0)) R package. Counts were normalized by Log2(TPM + 1) using the R package ADImpute (21 (v1.12.0)).

### Pathway activity calculation

Log_2_(TPM + 1) counts were used to calculate pathway activities using the PROGENy database (22), a compendium of publicly available signaling perturbation experiments based on footprint genes to yield a common core of 14 signaling pathways. Pathways regulatory activities were calculated using the Multivariate Linear Model (MLM) from the package decoupleR (23 (v2.9.7)).

### Immune cell-type deconvolution

In computational biology, deconvolution is an approach to quantitatively estimate the proportions of cell types in a mixed sample (e.g. bulk RNAseq) based on the observed gene expression profiles for separate cell types. Log_2_(TPM + 1) (transcript per million) normalized raw counts were used to estimate immune cell-type proportions for lymphocytes (B, T and NK cells), myeloid cells (monocytes, macrophages and dendritic cells) as well as cancer, endothelial, eosinophils, plasma, myocytes, mast cells and cancer-associated fibroblasts (CAFs). These cell-type proportion estimates were obtained by applying different reference-based deconvolution methods and several cell type signatures (see **Supplementary Table 1**). These methods can provide absolute cell abundance quantification using signatures derived from single cell and bulk RNA seq data.

### Transcription factor activity inference

Log_2_ (TPM + 1) counts were used to infer transcription factor (TF) activity. We use prior knowledge networks (PKN) to infer the activity of different TFs from the gene expression of its direct target genes quantified in the gene count matrix. We used CollecTRI (24) from the package decoupleR (23 (v2.9.7)), a collection of transcriptional regulatory interactions, which provides regulons containing signed transcription factor (TF) - target gene interactions compiled from 12 different resources as database and VIPER (25 (v1.30.0)) as the inference algorithm. Depending on the level of the counts and considering that one TF can have many targets and one target can be regulated by more than one TF, the algorithm can estimate the level of activity of the regulator based on correlation between gene expression values.

### Estimation of immune response scores estimation

Immune-scores were estimated on the TPM normalized raw counts using the EasieR package (26 (v1.4.0)) to generate immune profiles on a per sample basis. Briefly, immune-scores are calculated using gene sets that have been validated in different publications (see **Supplementary Table 2**) as signatures to estimate certain hallmarks of the immune response.

### Feature selection

The Boruta algorithm was applied using the R package Boruta (27 (v8.0.0)) using a bootstrapping approach to ensure consistency in the selection of features. Briefly, the algorithm performs feature selection and it was applied 100 times using different seeds, each time labeling features as ‘Confirmed’, ‘Tentative’ or ‘Rejected’. Features labeled as ‘Confirmed’ more than 90% of the times are finally selected.

### Processing of deconvolution features

Applying several combinations of deconvolution methods and signatures leads to several hundreds of features describing the TME landscapes in the samples. We applied specifically 6 methods (quanTIseq, XCell, MCPcounter, DeconRNASeq, EpidISH and CibersortX) and 9 signatures (BPRNACan, BPRNACanPro, BPRNACan3DProMet, TIL10, LM22, CCLE.TIL10, CBSX.HNSCC.scRNAseq, CBSX.Melanoma.scRNAseq and CBSX.NSCLC.PBMCs.scRNAseq), see **Supplementary Table 1** generating 351 features related to 13 cell types (and 30 subtypes). To reduce the dimensionality and eliminate redundancies we then applied a combination of unsupervised filtering techniques and iterative linear and proportionality based correlations within each cell type to form deconvolution feature subgroups. Applying an unsupervised approach, we removed features with a high proportion of zeros or low variance across samples. We then set out to eliminate redundant features calculating pairwise correlations of these filtered features to identify highly correlated (*≥* 0.7) feature pairs. We interpret these high correlations as evidence that those features are estimating the presence of the same cell-type despite potential differences in signature nomenclature and hence combine these features into a single feature subgroup. This procedure is carried out until no correlations above the specified threshold remain.

### Processing of TF activity features

The other set of descriptors of our samples stem from TF activity analysis, which returns a score of TF activity for each TF in each sample, amounting to 769 features. Adapting the Weighted correlation network analysis (WGCNA) approach (28 (v1.72-5)), we performed dimensionality reduction on these features by constructing what we defined as Weighted TFs co-activity networks (WTCNA) to detect highly correlated modules of TFs based on pairwise correlation of their inferred activity. Modules are defined as densely connected groups of nodes in the TF network, where connections represent correlation of activities, and they are arbitrarily named using colors. These TF modules were functionally characterized using pathway activities estimated for each sample (see Pathway Activity calculation above) and calculating the Pearson correlation between these TF module scores and the pathways activity scores. A PCA using the correlation matrix between the TFs module scores and the pathways activities allowed us to identify clusters of TF modules with correlated pathway activities, further grouping TF modules into broader functional groups. These combined TF module groups are named by combining the names of TF modules included, thus generating names that include multiple colors.

### TF modules functional enrichment analysis

TFs module enrichment was done by identifying the hub TFs from each module, these are genes which play a central role in the network’s module structure and function due to their high connectivity and influence on other genes. Thus, they often represent key regulators or drivers of important biological processes. We considered as hub TFs those which exhibit high module membership, meaning their activity is strongly correlated with the module’s score, indicating that they are highly representative of the module’s overall behavior. Also, since these genes are typically connected to many other genes within the network, we also considered the level of connectivity for the hub selection. We selected TFs with a high “degree”, a measure of the number of direct connections or edges a TF has with other TFs in the network. Overall, TFs with high module membership (r>0.8) and belonging to the top 10% of genes with high degree were selected as hub TFs. From the hub TFs, we identified their corresponding target genes using the CollecTRI database (24). We considered only the top 20% most variable (based on gene expression) and unique target genes per TF module. Using these lists, we performed an over representation analysis (ORA) using the R package ReactomePA (29 (v1.46.0)) and the Reactome database (30 (v1.86.2)) to provide functional interpretation of the modules.

### Integration of deconvolution and TF features

Using both deconvolution and TF activity features across samples we set out to define combined features as groups of cells that share TF activity profiles, potentially describing their phenotypic states. We performed hierarchical clustering using ward.D2 as the agglomeration method of the matrix of correlation between grouped deconvolution features and TF module scores. This leads to clustering of grouped deconvolution features that each refer to specific cell types, producing further grouping of *different* cell types. We refer to these as Cell type groups. The existence of these cell type groups suggests that several cell types could be activating specific biological processes, as reflected by similar activities of the TF modules, potentially revealing different cell states (e.g. cell growth profiles could be observed in cancer cells or fibroblasts by detecting similar TF activities across patients). These new cell type groups are new features composed of different grouped deconvolution features referring to different cell types, named using a specific nomenclature (e.g Dendrogram_red_turquoise.group_2) where “dendrogram” indicates that the feature came from a hierarchical clustering, colors indicate which TFs module were merged to produce the dendrogram and “group_x” refers to the actual cluster in this dendrogram. This definition of cell type groups implies cell types cluster together because they share similar biological activity as measured by the TFs activity profiles. Finally, the Boruta feature selection algorithm (see above) was applied to measure the importance of these integrated features in the classification of samples.

### Validation cohort

An independent cohort consisting of 77 surgically resected adenocarcinomas (1 = stage 0, 44 = stage I, 26 = stage II and 6 = stage III) (31), from which only 70 early stage (I, II) samples were used to validate the findings from the primary analysis cohort. The validation cohort samples were collected at Vanderbilt University Medical Center, Nashville, TN, from treatment-naive patients undergoing surgical resection. The dataset included both bulk and scRNAseq samples, with 15 patients for this last one. From this, only 9 patients have both bulk and scRNAseq information.

### Single-cell RNAseq analysis

Preprocessed single-cell RNAseq data was obtained from (31). The Seurat package (6, 32–34 (v4.3.0.1)) was used for downstream analysis of the data in the R environment. We computed a principal component analysis for dimensionality reduction followed by the neighborhood graph on the first 20 principal components, using the elbow plot, obtaining 24 clusters. Cell annotation was done with the following references: Human Primary Cell Atlas, Immune Cell Expression, Monaco and Blueprint Encode using the celldex R package (35 (v1.10.1)). A consensus of all three annotations was taken for identification of NK clusters. Reference-based deconvolution was done using the scRNAseq object and the BayesPrism method (36 (v.2.0.0)), obtained from the Omnideconv R package (37 (v.0.1.0)).

### Survival analysis

Patients from the validation cohort with early stage disease (Stage I and II) were included in a survival analysis. “Time” is measured in days and “event” was defined as either death, recurrence or progression. Cox proportional hazards modeling was performed using the R packages rms (v.6.8.0) and survival (38, 39 (v3.5.5)), and Kaplan-Meier curves were prepared using ggplot2 (40 (v3.4.3)) and Survminer (41 (v0.4.9)). Univariate and multivariate cox proportional hazards (coxPH) models were evaluated across selected cell type groups to investigate whether the effect of a single or multiple cell type groups on the hazard of an event (death/progression/recurrence) was significant for the survival outcomes. After fitting the CoxPH models to different cell type groups combinations, we stratified our patients based on the linear predictors of the model (risk scores) from which we define as ‘high’ the patients with risk scores above the median value of the cox model’s linear predictors and as ‘low’ the patients below it. We then performed a Kaplan Meier analysis and plotted the survival curves for each risk group. Finally both survival curves were assessed via a log rank test to see if there was a statistically significant difference between risk groups (p value < 0.01).

### TCGA analysis

Samples counts from TCGA were retrieved using TCGAbiolinks R package (42–44 (v2.30.4)). We selected open-access cases from the project TCGA-LUAD, using transcriptome profiling as data category, RNA-seq as experimental assay and STAR-counts as analysis workflow type. Applying these filters, 600 cases were retrieved. Since our focus was only on early stage samples (I, II) we selected the corresponding 399 cases. Survival analysis was done following the same pipeline described above; for this, the variables “vital_status”, “days_to_last_follow_up”, “days_to_death” were considered to determine the overall_survival (PFS) and whether the event (death) occurred. Six patients were removed from this analysis due to the presence of missing values in these variables.

## Results

### The Lung Predict cohort

**Figure 1:**
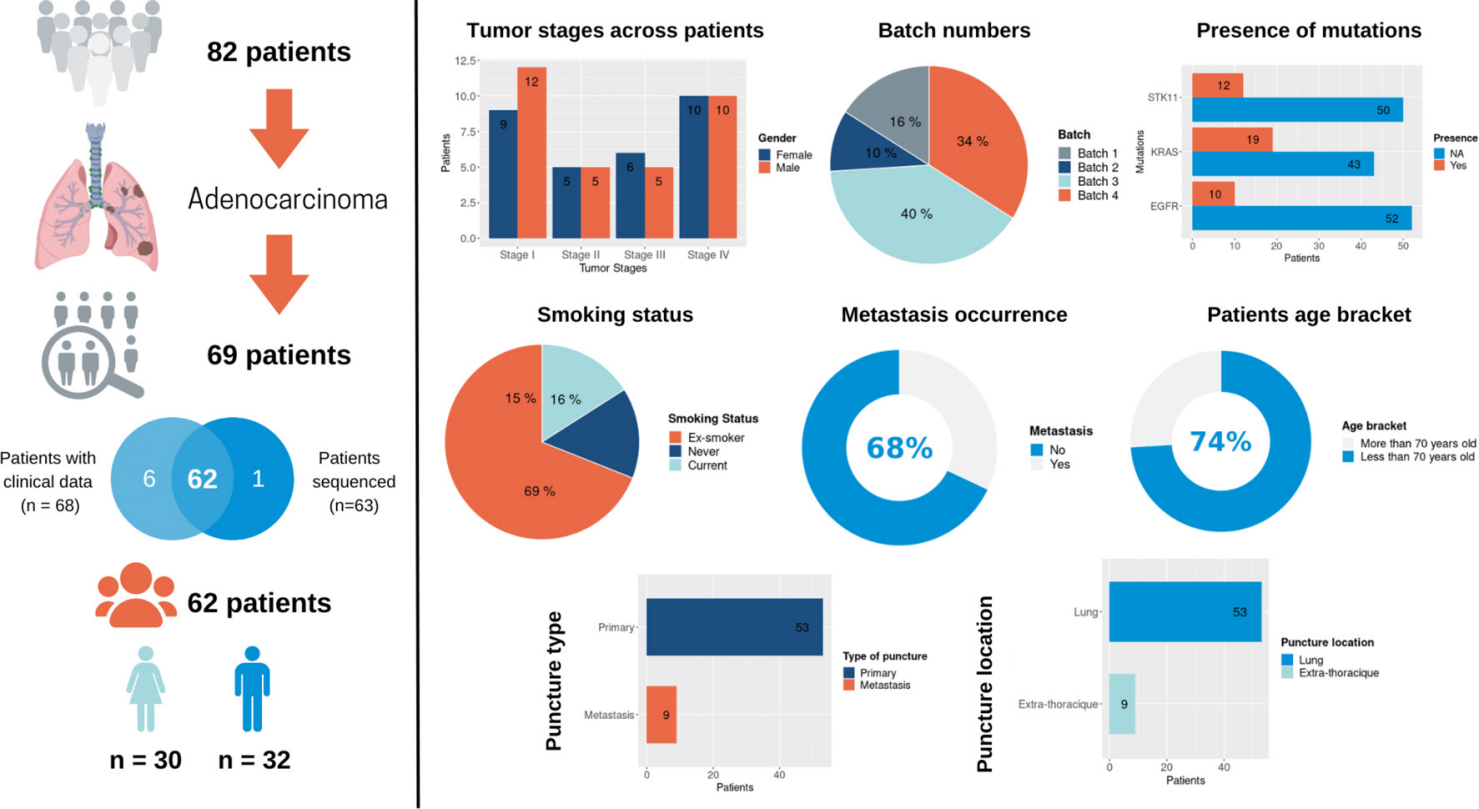
An overview of the Lung Predict cohort. A description of the cohort is presented on the left with some summary graphics on the right specifically detailing tumor stages in male and female patients, RNAseq batches, presence of the most frequent somatic mutations as detected by a gene panel assay (STK11, EGFR, KRAS), smoking status, metastasis occurrence, age category, type of sample (primary or metastatic sample), location of the sample.

Bulk RNAseq was performed on surgically resected tumor tissue or tumor biopsies from 82 patients with NSCLC, 62 of which were diagnosed as lung adenocarcinoma (LUAD) and were considered further (the “Primary Analysis Cohort”). Of these 62 adenocarcinomas, 30 are female and 32 are male; 21 were enrolled at Stage I, 10 at Stage II, 11 at Stage III and 20 at Stage IV. A full breakdown of this cohort is presented in **Table 1** and a patient inclusion flow chart is included in **Figure 1**, together with details of the validation cohort (see the following sections).

**Table 1:** Summary of the total number of patients included in the Lung Predict and the Vanderbilt validation cohort (VUMC) (percentages of totals in brackets).

	Lung Predict		VUMC	
**Total**	62		77	
**Sex (Female)**	30	*(48)*	42	*(55)*
**Age (<70)**	46	*(74)*	47	*(61)*
**Smoking Status: Never**	10	*(16)*	14	*(18)*
**Smoking status: Former**	9	*(15)*	53	*(69)*
**Smoking status: Current**	43	*(69)*	10	*(13)*
Stage				
0	–	*-*	1	(1)
I	21	*(34)*	44	*(57)*
II	10	*(16)*	26	*(34)*
III	11	*(18)*	6	(8)
IV	20	*(32)*		-
**Metastatic (non-primary)**	20	*(32)*	0	–

We applied a computational immunology approach integrating several features derived from transcriptomics data to better characterize and profile the TME of LUAD tumor samples in our cohort. The features extracted included cell-type proportions, level of activity of specific Transcription Factors (TFs) and scores of immunogenicity commonly used in the literature.

Briefly, reference-based deconvolution involves applying statistical methods to infer cell type proportions in biological tissue samples starting from transcriptomic profiles of specific cell types (signatures) and bulk transcriptomics from the samples, such as tumoural tissues in this case. We applied several deconvolution methods to the transcriptomes from our LUAD samples and used different cell type signatures to generate estimates of cell type proportions (see Methods, see **Supplementary Table 1**).

Normally, the application of dimensionality reduction methods, such as pathway activity analysis or the calculation of immune cell type proportions allows better interpretation of the signal from bulk gene expression data but at the cost of introducing artificial noise or removing potentially interesting data features. Selecting deconvolution methods is not trivial and the different results obtained with different methods and signatures suggest that they capture different aspects of the samples. In this study, we aimed to use a variety of different methods and signatures instead of choosing a single one. However, using several deconvolution methods and signatures, each covering a range of cell types, produced over 300 different deconvolution-related features, which led us to an increase in dimensionality, exposed high variability between features related to the same cell types, ultimately hindering interpretation and imposing the need for much larger sample sizes to achieve statistical power. This pushed us to address these issues by engineering novel approaches to produce meaningful cell deconvolution features integrated with TF activity profiles.

We therefore performed TF activity estimation, which is an approach to quantify the strength of activity of specific TFs (essentially an estimated combination of their abundance as proteins and their post-translational modifications if required for their activity) based on the expression level of their targets. These approaches involve a prior-knowledge network of TF-target interactions in combination with gene expression levels from bulk transcriptomics data and they allow to identify activation of specific regulons (TFs and their targets) despite the fact that TF activities are rarely regulated at the transcriptional level (see Methods). Complementary to this analysis, we have calculated scores of activation of specific pathways using PROGENy, which help us to define the processes that dominate the transcriptome of our patient samples (see Methods).

Finally, several scores have been proposed in the literature to estimate the level of immunogenicity in tumor samples from bulk transcriptomics data and we have calculated these immuno-scores across our cohort (see Methods).

### Deconvolution features along with inferred TF activity profiles reveal different immune profiles describing the tumor microenvironment across patients of different stages

As a result of applying cell type deconvolution, we considered 351 features for each sample. Multiple signatures were used for each cell type, leading to several estimates of proportions of the same cell type (e.g. monocytes). This multiplication of features referred to the same cell type is due either to the fact that they capture different cell subtypes (e.g. classical or non-classical monocytes), or simply to differences in the generation of the signatures from the literature (from *in-vitro* co-cultures, from tumor samples, etc.). To reduce the redundancy and dimensionality in our data we first grouped deconvolution features quantifying the same cell types based on their correlation across patients and generated deconvolution feature subgroups (see Methods). Briefly, if multiple features estimating the same cell type have high correlation it suggests that they do not differ biologically and do not capture distinct subtypes, so we merge the corresponding features) (see **Supplementary Table 3** for details).

**Figure 2:**
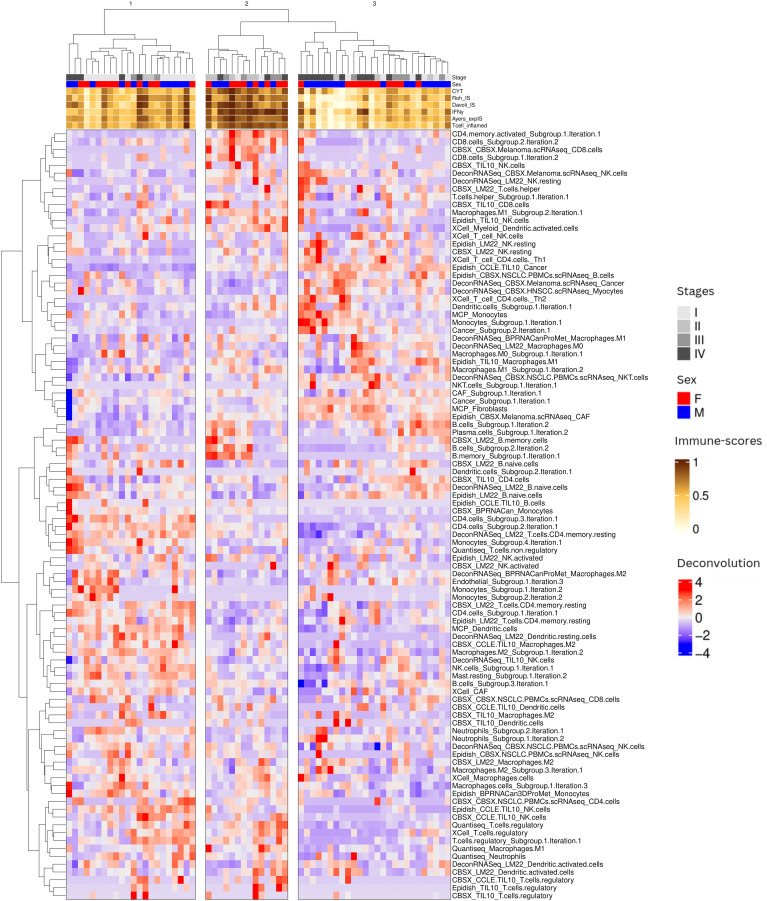
Overview of patient sample clustering based on immune deconvolution subgroups. Our immune deconvolution features after being processed identified three clusters of patients corresponding to “cold/intermediate”, “hot” and intermediate tumors. Heatmap showing patients within the 3 clusters identified by hierarchical clustering. The gray scale at the top corresponds to the stage of the disease: the darker the color, the later the stage. The orange to brown scale corresponds to the immune-scores (see Methods): the darker the color, the higher the immune scores.

We then performed unsupervised hierarchical clustering of patient samples based on the grouped deconvolution features, to identify patient clusters with correlated immune cell proportion. We identified three patient groups based on the grouped deconvolution features and interpreted them based on the immuno-scores in each sample. Patient cluster 1 contained mostly “intermediate” tumors, patient cluster 2 contained mainly “hot” tumors and patient cluster 3 was constituted by a mixture of “cold/intermediate” tumors (**Figure 2**). We also visually notice more early stage samples (I, II) in patient cluster 1, a high presence of late stage samples (IV) in patient cluster 3 and a high presence of intermediate stages (III) in patient cluster 2.

To estimate the main processes driving the transcriptomic profiles of our samples, we calculated TF activities and constructed weighted co-activity TFs networks, identifying TF modules (named with colors), which are groups of TFs showing correlated activity profiles across samples (see Methods and **Supplementary Table 4** for TF module composition). We then applied a Boruta feature selection approach to select the most important deconvolution features driving the patient classification into these three patient clusters, identifying 27 deconvolution features to be the most influential.

**Figure 3:**
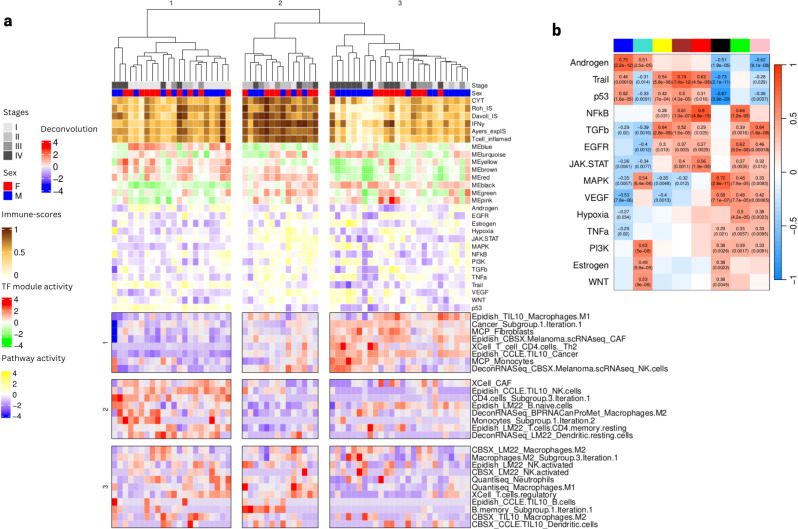
Annotation of the three patient clusters from (**Figure 2**) using TFs module scores, pathways scores, and values of Boruta selected immune deconvolution features. **(A)** Three feature groups (in rows) are identified from the deconvolution features (values shown on scale red to blue from high to low). The panel also shows as column annotations of each sample the immuno-score (brown to white from high to low), the TF module scores (red to green from high to low, see composition of each module in (**Supplementary Table 4**) and the pathway scores (yellow to blue from high to low). **(B)** Heatmap showing significant Pearson correlation between pathway activities and TF modules scores shown in panel A (denoted by the colors at the top of the columns: blue, cyan, yellow, brown, red, black, green, pink from left to right). Heatmap colors represent levels of correlation (darker red implies high positive correlation, darker blue implies high negative correlation). Statistics are shown as text only for significant correlations (p value < 0.05).

To further investigate the mechanistic processes that might underlie the 3 patient clusters, we observed how different features (cell type composition, TF module scores, and pathway scores) correlated with each other across patients (c.f. **Figure 2**). We note that several cell types appear in multiple rows as separated features, possibly indicating that the different signatures capture distinct cell subtypes. For example we observe multiple features related to NK cells and B cells. The name of the feature reflects the name of the public signature that generated this feature and often suggests which subtype is captured (activated/naive etc.), while the names including ‘subgroup’ denote several features that were combined in the earlier step of deconvolution feature grouping since they displayed strong correlations across patients. We characterized the 3 patient subgroups as follows (**Figure 3**) according to the values of different cell type features:

*Patient cluster 1 (“intermediate” tumors):* associated to low presence of cancer cells, several CAF signatures, some myeloid cells (M1 macrophages and monocytes), some lymphocytes (CD4 T helper), some type of unspecified NK cells and higher abundance of B cells, resting CD4 and dendritic cells with NK cells denoted as activated. TF activity analysis showed an involvement of TFs modules yellow, brown, red and blue, involved in biological pathway activities related to Androgen, Trail and p53, suggesting a relation with apoptosis and tumor suppression.

*Patient cluster 2 (“hot” tumors):* associated to intermediate presence of cancer cells, several CAF signatures, some myeloid cells (M1 macrophages and monocytes), some lymphocytes (CD4 T helper) and some type of unspecified NK cells and low abundance of naive B cells, resting CD4 and dendritic cells and NK cells denoted as activated with varying levels of a group of non-naive B cells. TF activity analysis showed high scores in modules black, green, red and brown, which seems to be related to high scores of JAK/STAT, VEGF, MAPK and hypoxia pathways, as well as low levels of modules blue, and yellow, with particularly low scores for Trail and p53, suggesting activation of immunity, stress response and proliferation.

*Patient cluster 3 (“cold/intermediate” tumors):* mostly late stage, showing particularly high proportions of cancer cells, CAF cells and some macrophages. TF activity showed particularly high scores for module black and low scores for Trail, p53 but also NFkB, VEGF and JAK/STAT, MAPK, suggesting a highly aggressive, immunosuppressive and proliferative phenotype.

To better interpret the duality of specific cell type features we consider how they correlate with each other (row feature groups 1 to 3 on **Figure 3**).

Interestingly, we identified a complex behavior profile for the different features estimating the presence of the same cell types. In some cases, signature names can suggest which cell subtype we are considering but there are known issues with signatures for myeloid cell subtypes, for example, despite the importance of these details to understand whether the TME is immunosuppressive or not. Here we focus on NK cells, for which several signatures appear to give conflicting results. The first NK profile (exemplified by the *NK CBSX_melanoma …* feature from feature group 1) is associated with a presence of cancer cells and CAFs and is found in samples with lower immune scores (subset of Patient cluster 3). This profile may imply the presence of dysfunctional NK cells, which are characterized by reduced proliferation and cytotoxic capabilities. The other profile (NK from *EpiDISH_CCLE_NK …* from feature group 2) is associated with endothelial cells and the presence of certain B and CD4 T-cells found in samples with intermediate immune-scores, perhaps signifying the presence of tertiary lymphoid structures (TLS), organized immune cell aggregates that can be good prognosis markers when identified through spatial omics. Another group of NK cells (NK from feature group 3) more associated with the presence of neutrophils, dendritic and M2 macrophages does not seem to be associated to the 3 patient clusters identified, showing variable values in all sample clusters, Taken together, these findings suggest that NK cells of different kinds, associated with other immune cells, can be found in either immune-desert tumors, where they are likely to be dysfunctional, typically in late stage samples, but also in intermediate tumors and early stage samples, where they can be associated with different kinds of immune landscapes, depending on their partners.

### Data integration to uncover associations between cell-type deconvolution features and TF activity profiles

Having observed interesting associations between TF activity and pathway scores and TME landscapes, and the duality of certain cell types (NK for example) we decided to investigate whether a combination of these features could reveal connections between cell states across the different cell types in the TME. As an example, we reasoned that the presence of specific cytokines in the tumor could have an impact on the state of specific immune cells (say cytotoxicity of NK cells) in specific patients. We therefore set out to develop a computational method to integrate cell type proportion estimates and TF activity scores to evaluate the state of the different cell populations present in the samples.

**Figure 4:**
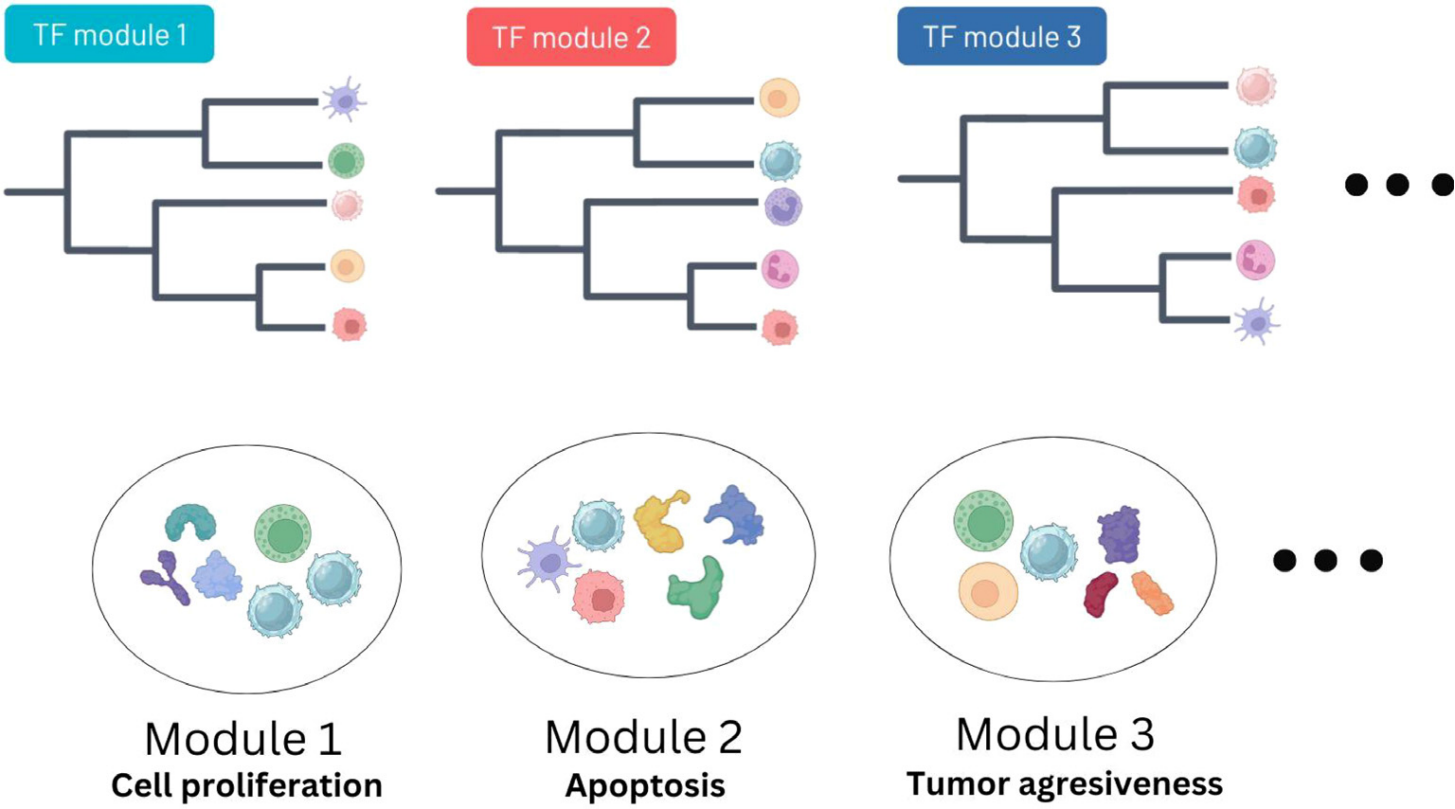
Overview of the deconvolution and TF activity integration pipeline. Immune cell deconvolution features and modules of TFs sharing inferred activity profiles are integrated together using a combination of clustering methods in order to reduce the dimensionality of the results (see Methods). The output are groups of immune cells characterized by the same TF activities.

Briefly, we start by considering the grouped deconvolution features and TF module activity scores as descriptors of our samples. Since TF module scores and grouped deconvolution features can both be calculated in each sample, we can visualize the association between each TF module and the different grouped deconvolution features as a heatmap. Hierarchical clustering of this matrix (deconvolution feature by TF module) allows us to cluster deconvolution features, even grouping those that estimate proportions of different cell types, allowing us to define Cell Type Groups (see Methods). The appearance of clusters of deconvolution features referring to different cell types but having similar TF activity profiles suggests some commonality of biological processes ongoing in the distinct cell types present in specific patients (**Figure 4**).

### Integrated analysis of cell type composition and TF activity profiles in early stage LUAD samples uncovers two distinct patient groups

Our results highlighted a possible difference in the immune profile of samples according to stage, with most late stage samples (stage IV) being in ‘cold’ patient groups. To avoid any confounding effect of stage and sample type (primary vs. metastasis biopsy) and reduce the heterogeneity of processes likely to take place in our samples, we decided to focus on the early stage samples (stages I and II).

To assess the immune landscape in the stage I and II samples of the Lung Predict cohort, we performed immune cell-type deconvolution and inferred TF activities across these samples (see Methods).

Focusing specifically on stage I and II from the Lung Predict cohort (31 samples), we applied our integrative approach to combine grouped deconvolution features and TF module activity scores. (99 deconvolution features including 46 cell subgroups (**Supplementary Table 5**) and 53 non-grouped features, 7 TF modules (**Supplementary Table 6**), each containing different groups of TFs (**Supplementary Figure 1A**) correlating with different biological activities (**Supplementary Figure 1B**).

In order to further study the composition of these modules, we identified the most central (hub) TFs in each module (see Methods), which highlighted 20 hub TFs in total (6 for module red, 6 for module brown, 3 for module black, 2 for module green and 3 for module blue). No hub TFs were found for module turquoise and yellow (**Supplementary Figure 1C**). Further enrichment of these TFs modules was done by identifying the corresponding target genes of the hub TFs (see Methods). Using only target genes that belong to only one module to avoid overlapping ones, we performed an over-representation analysis (ORA) and identified enriched pathways using the Reactome database. Results showed an enrichment for neutrophil degranulation and chemokines binding for TFs module black, suggesting a potential role of this module in the interaction of neutrophils with other cells. The Brown module is mostly enriched in pathways related to EGFR signaling, suggesting a potential role of these TFs in regulation of cell growth. Module blue showed enrichment for toll-like receptor pathway components, suggesting an association with immune suppression factors and tumor progression. Module green showed an enrichment in transmembrane transporters, this might suggest the metabolic uptake and efflux of nutrients and the metabolic crosstalk between cells in the TME. Finally, module red is enriched in cell cycle checkpoint terms, confirming its role in regulation of cell proliferation (**Supplementary Figure 1D**).

In order to reduce the dimensionality of these TF modules, we identified different module categories by using information of signaling pathways from PROGENy (see Methods). From this we performed a PCA analysis to see which TF modules cluster together based on their association with these pathways. TF modules blue, green and yellow clustered together and showed a common activation of p53 and apoptosis pathways while TF modules black, brown, turquoise and red grouped together by showing a similar association to VEGF, NFkB and TGFb (**Supplementary Figure 2**).

Taken together and considering both enrichment and PCA analysis, our results showed that modules blue, green and yellow are more associated with cancer-related pathways, including tumor suppression and progression; while brown, black, turquoise and red have an association with cell growth.

**Figure 5:**
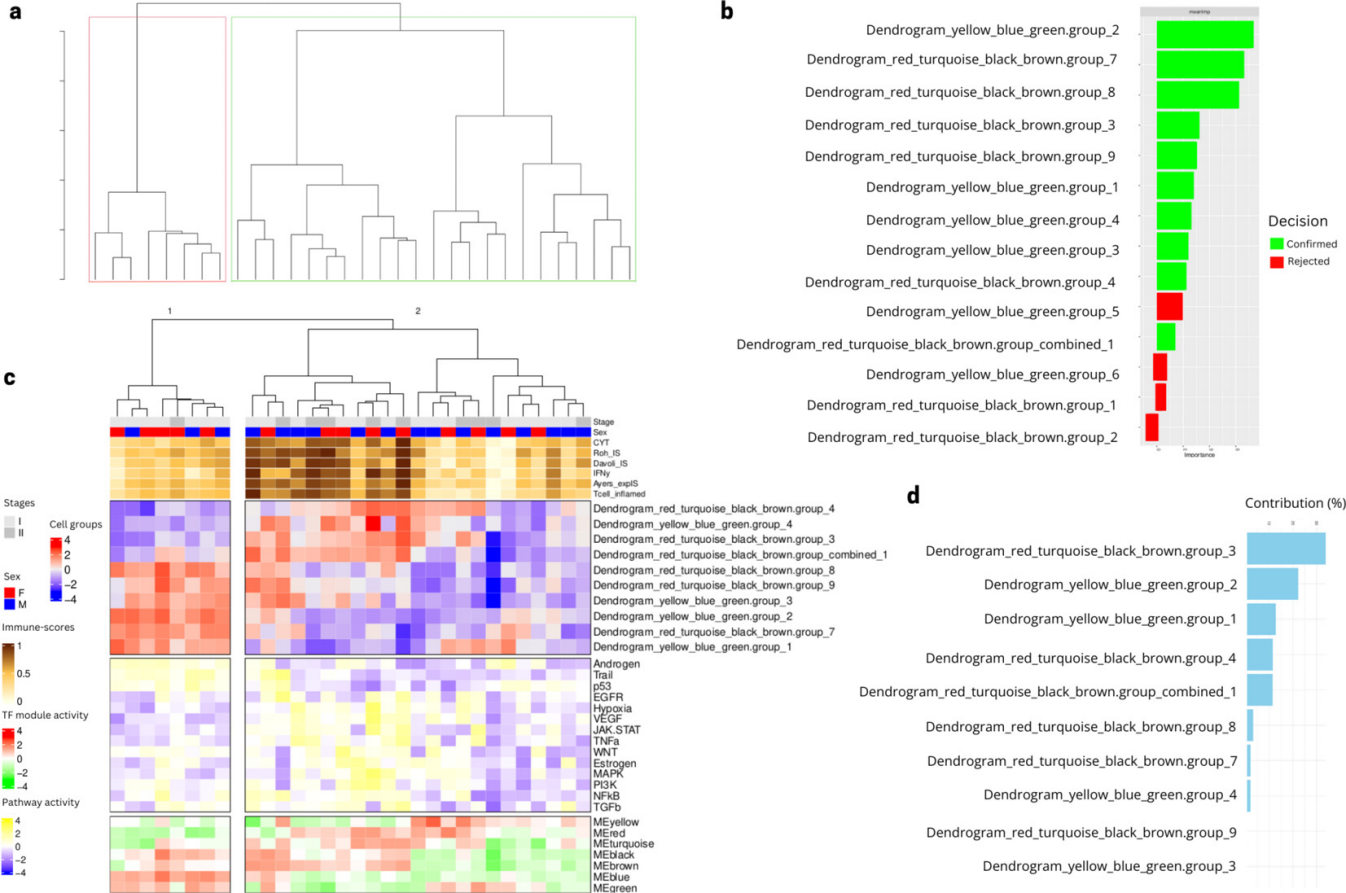
Selected cell type group features reveal two profiles in the Lung Predict early stage cohort. **(A)** Hierarchical clustering dendrogram of early stage patient samples using the 14 cell type group scores. **(B)** Feature selection based on importance for predicting the two patient clusters in 5A using a Boruta algorithm, showing confirmed features (green) and rejected features (red) after 100 repeats (see Methods). **(C)** Heatmap showing the 10 cell groups selected after feature selection. The panel also shows as column annotations of each sample the immuno-score (brown to white from high to low), the TF module scores (red to green from high to low, see composition of each module in **Supplementary Table 5**) and the pathway scores (yellow to blue from high to low). **(D)** Contribution of cell type groups to the PCA variation in the classification of patient clusters.

Associations between these two categories of TFs modules and deconvolution features were investigated defining several cell type groups with correlated TF module scores (see Methods). As a reminder, cell type groups consist of subsets of grouped deconvolution features that share similar TF profiles, for example, Dendrogram_red_turquoise_black_brown.group_1, which contains several deconvolution features related to B cells, cancer cells and dendritic cells (**Supplementary Table 7**). With this approach, 14 cell type groups containing deconvolution features with significant Pearson correlations with TF module scores (p-value < 0.05, cut.height = 5) were identified. These cell type groups naturally divide patients into two groups (**Figure 5A**). A feature selection algorithm (see Methods) was applied to estimate the importance of different cell type groups in the classification of patients in the two patient clusters identified by unsupervised hierarchical clustering. After 100 repeats, 10 cell type group features were selected as important to classify Lung Predict early stage samples into the two patient clusters shown in (**Figures 5A, B**). These cell type group features can themselves be grouped into two main broader categories (**Figure 5C**).

Performing a PCA using these cell type groups as features across the samples, we observed that two cell type groups (PCA variance explained >20%) were mostly driving this separation of the two patient groups (**Figure 5D**). The first, namely “Dendrogram_red_turquoise_black_brown.group_3”.

is composed mainly by resting NK cells, cancer cells, fibroblasts, CAF, NKT cells, T helper cells, dendritic cells and M1/M0 macrophages (**Supplementary Table 7**) and is significantly associated with pathways related to cell growth and angiogenesis based on the TF modules involved (red, turquoise, black and brown, c.f. **Supplementary Figures 1B, C**).

The second cell type group, namely “Dendrogram_yellow_blue_green.group_2”, is highly present in patients with intermediate immune-scores and is composed mostly by CD4 T cells, dendritic cells, M2 macrophages, neutrophils, monocytes, mast cells, endothelial cells and NK cells, while being associated to pathways related to immune response activation and tumor suppression based on TF modules involved (yellow, blue, green, c.f. **Supplementary Figures 1B, C**).

### The two patient subgroups identified in the LungPredict early stage samples are validated in an external early stage LUAD cohort

Senosain et al. have recently published an in-depth characterisation of an early stage clinically annotated LUAD cohort (31). This cohort, to which we will refer as Vanderbilt, including 70 early-stage (stage I and II) lung adenocarcinomas, for which bulk RNAseq as well as 15 scRNAseq samples are available (with an overlap of 9 patients), was used as external validation.

Before using the validation cohort, we verified that these two datasets Lung Predict and Vanderbilt were comparable (**Supplementary Text 1**, **Supplementary Figures 3**, **4**).

**Figure 6:**
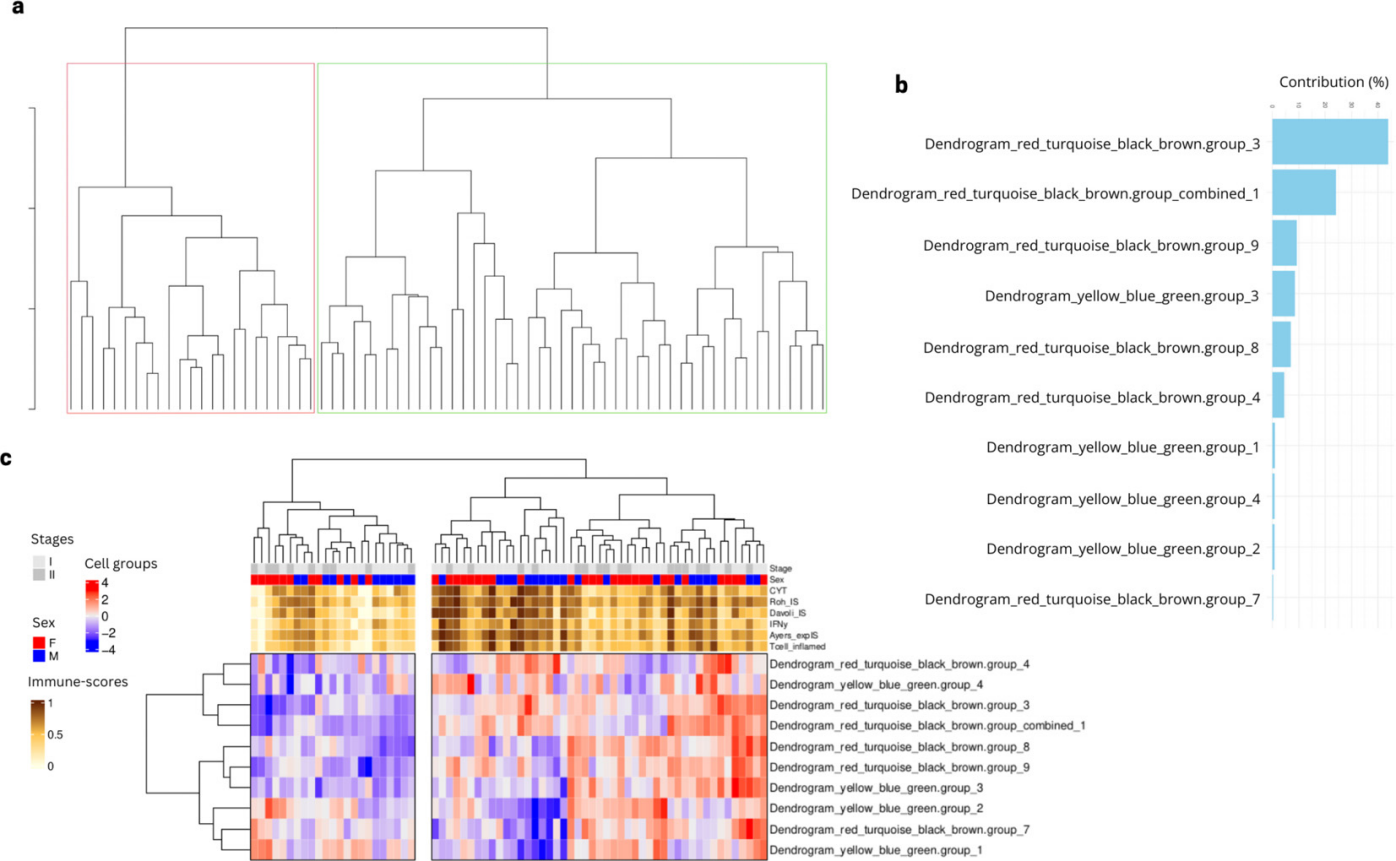
Selected cell type groups features found in LP cohort projected in early stage samples of validation cohort. **(A)** Dendrogram obtained by hierarchical clustering revealed two groups of patients based on the cell type groups values. **(B)** Cell type groups feature contribution to the PCA variation in the classification of patient clusters. **(C)** Early stage patient samples from the validation cohort are divided in two main clusters based on the values of the selected cell type groups from LP analysis.

To validate our newly identified patient clusters, we considered the same 10 most important cell type groups identified via the feature selection algorithm using data from our validation cohort to see if the identified groups can also classify an independent cohort, namely the stage I and II samples from the Vanderbilt cohort. We performed a cell group projection analysis, which consists of identifying the same TFs modules based on the gene expression from the independent cohort. We then projected the same deconvolution subgroups into the unprocessed deconvolution features from the Vanderbilt samples and recreated the same cell type groups identified in the Lung Predict cohort. The independent validation cohort samples also display a separation into two patient groups based on the values of the selected cell type groups (**Figure 6A**). A PCA analysis suggests that the feature with the highest contribution (>40%) is the same as in the Lung Predict analysis (**Figure 6B**). This important cell type group is composed mainly by resting NK cells and M1 cells and associated with cell growth and angiogenesis. This cell type group is present mostly in patients with intermediate and high immune-scores and lacking in patients with low immune-scores (**Figure 6C**).

Taken together, these results suggest that the two patient groups we identified in the LP cohort are also identified in the validation cohort. Our findings suggest the importance of resting NK and M1 cells and activation of cell growth and angiogenesis in the separation of the two patient clusters observed similarly in the two cohorts.

### Differential expression analysis between patients with alternative profiles of NK cells hints at differing cytotoxicity of these cells

**Figure 7:**
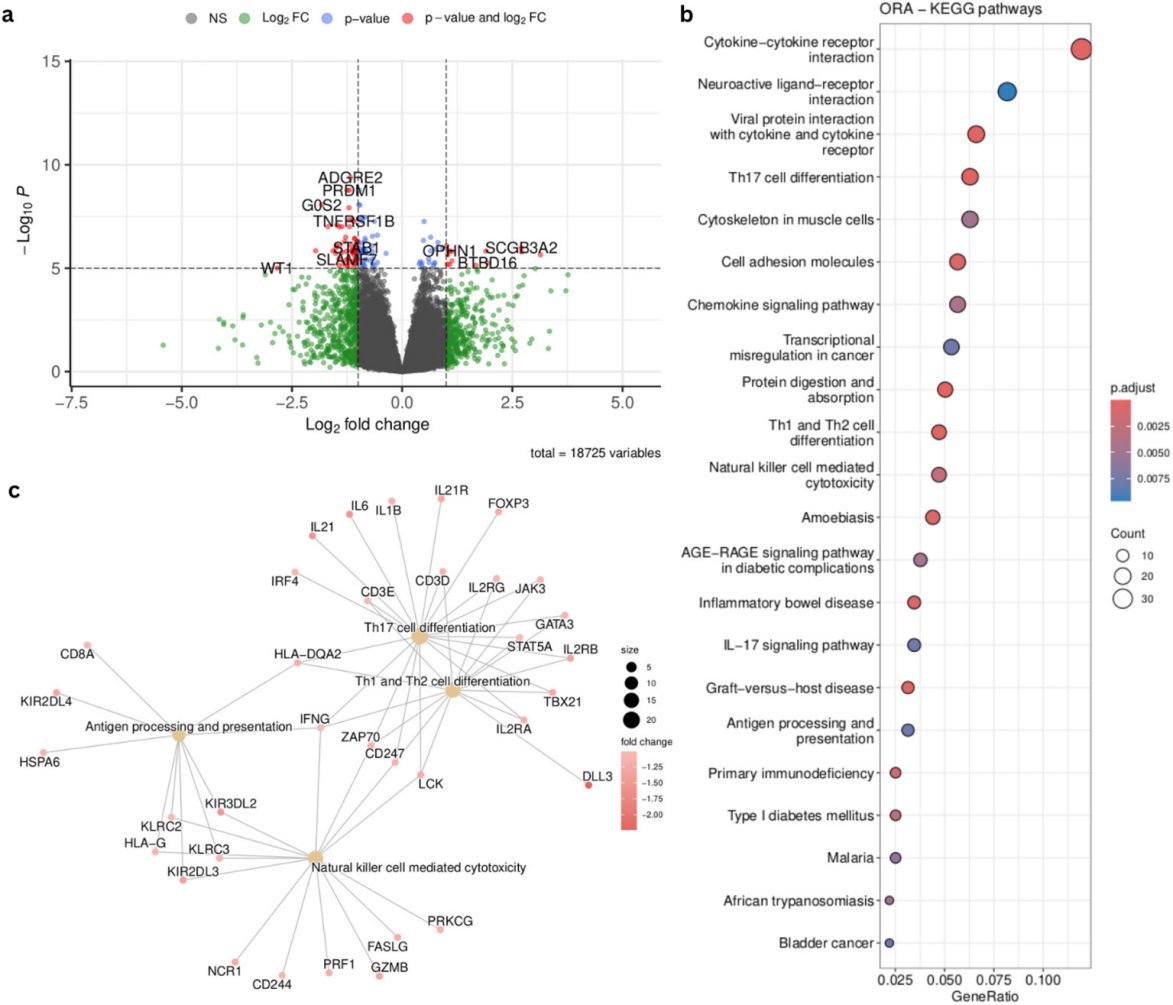
Supervised analysis of the patient groups identified in **Figure 6A**. **(A)** Volcano plot summarizing the 665 differentially expressed genes (DEGs) (padj < 0.05, abs(log2FoldChange) > 1) identified by comparing the green and red clusters from **Figure 6A** **(B)** Top results from the KEGG pathway enrichment analysis (p value < 0.01) on the DEGs summarized in the volcano plot. **(C)** Network plot of immunological pathways showing the genes involved in each pathway and overlapping among pathways. Node colors communicate the log2FoldChange of the genes between the two patient groups.

In order to understand the difference between two clusters of patients defined by the selected cell type groups, we performed a differential expression analysis between Vanderbilt patients from cluster 1 (green) and Cluster 2 (red) in **Figure 6A**. We obtained 665 differential expressed genes (p.adj < 0.05, abs(log2FoldChange) > 1) between the two patient subgroups (**Figure 7A**). We then summarized these DEGs into KEGG pathways identifying enrichment of deregulated genes in several immunologically and oncologically relevant pathways (p value < 0.01) (**Figure 7B**), including the NK cell-mediated cytotoxicity pathway. A network plot was generated linking enriched pathways and the genes contained in them in order to interrogate the genes present in this pathway and understand the overlap with other immunologically relevant pathways (**Figure 7C**). In this network plot, we see the downregulation of CD3 (epsilon and delta) as well as CD8 alpha suggesting a reduction in the activation - and, perhaps, no involvement - of CD8 T-cells in the functional profile of NK cells from cluster 1. Many KIR genes, important for NK cytotoxicity) appear downregulated in this cluster, confirming the potential presence of dysfunctional or resting NK cells in this first cluster of patients. In a deeper analysis of the NK-cell mediated cytotoxicity pathway (**Supplementary Figure 5**), we observe a downregulation in inhibitory receptors KLRC1 (NKG2A) and KIR3DL2. The inhibitory potential of NKG2A is dependent on its dimerization with CD94, which is not differentially expressed in our analysis (45). Anfossi et al. reported that KIR+NKG2A+ NK cells were responsive upon stimulation with tumor targets whereas NK cells lacking these inhibitory markers are hyporesponsive (46). Furthermore, the observed downregulation in protein kinase C (PKC) can have a direct effect on the granulization and cytotoxic effect of these NK cells (47). Taken together, these results suggest that these two patient clusters might be defined by the presence of either functional or dysfunctional (resting) NK cells.

### Single-cell analysis in the validation cohort confirms multiple subgroups of NK cells

**Figure 8:**
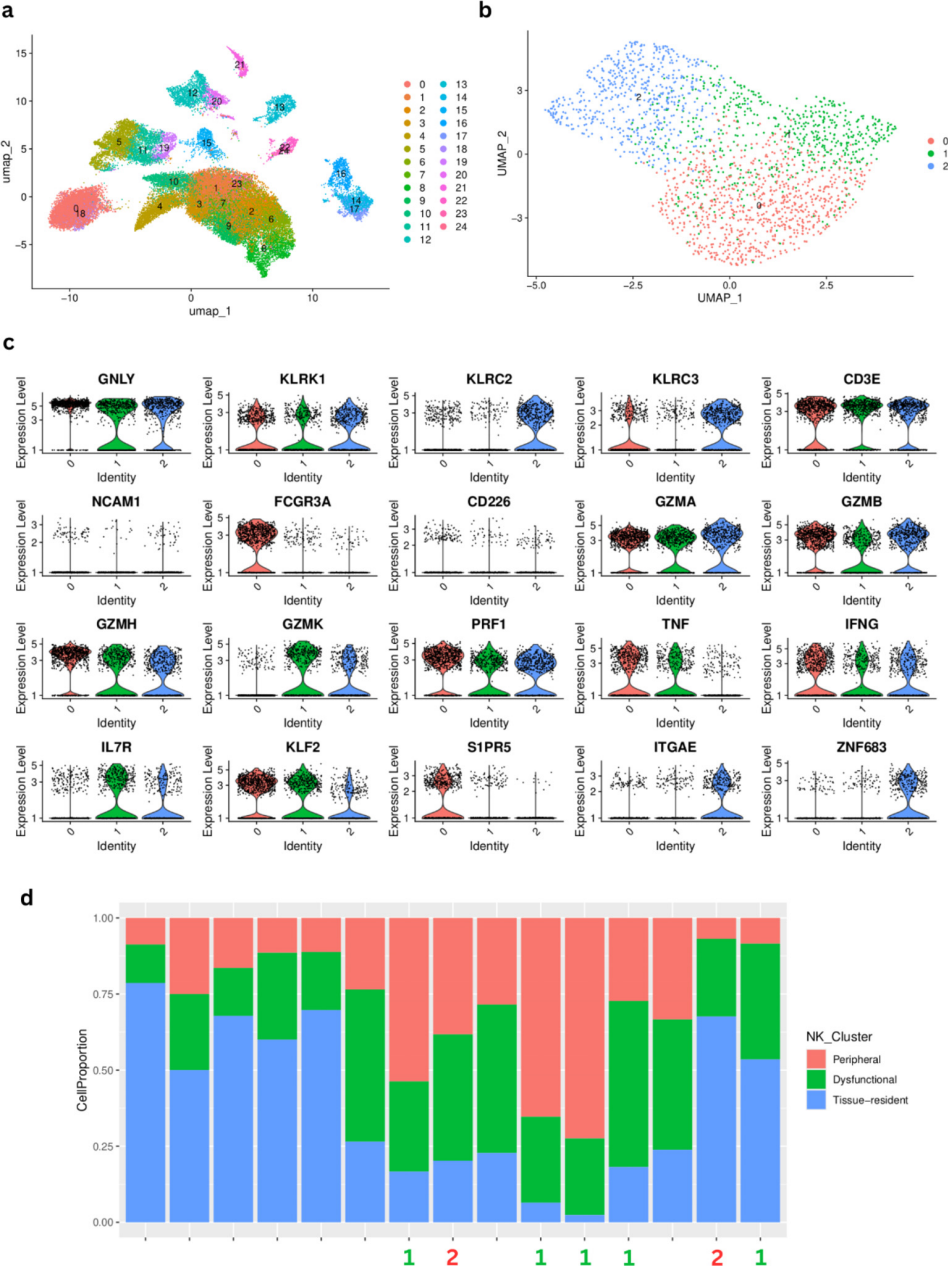
Single-cell RNAseq characterization of natural killer (NK)-cell clusters in LUAD samples from the Vanderbilt cohort. **(A)** Graph-based UMAP clustering. **(B)** UMAP of cluster 8 identified as NK cells after automatic annotation showing the 3 NK subclusters. **(C)** Characterization of the three NK subclusters using several cell surface markers. **(D)** Proportions of each NK cluster, labeled according to the marker analysis. The numbers at the bottom correspond to the patient cluster to which the corresponding bulk RNAseq sample belongs (Cluster 1= green, Cluster 2 = red) according to (**Figure 6A)**.

In an effort to better characterize the dual behavior associated with NK cells detected at the bulk RNAseq level, we analyzed single cell transcriptomics data from 15 patients from our validation cohort. Following standard procedures for scRNAseq analysis, we performed graph-based clustering of cells to identify cell groups sharing similar gene expression (**Figure 8A**) using annotations already provided in the scRNAseq object from the validation cohort (31) and didn’t identify any batch effect (Supplementary Figure 6). Since our focus was to identify different NK subclusters, we then re-annotated these cells. We performed annotation using reference expression datasets with curated cell type labels for automatic annotation in order to establish a consensus for the NK cell annotation (see Methods) (**Supplementary Figure 7**). We extracted the cell clusters identified as NK (cluster 8) and performed an additional clustering step to identify subclusters within this population. We obtained 3 subclusters of NK cells (**Figure 8B**) that we investigated based on specific NK markers. All three subclusters showed a high expression of KLRK1, which is expressed on all NK cells as well as on a small subset of cytotoxic CD8 T-cells. Interestingly, when profiling the expression of GNLY (cytolytic compound expressed by cytotoxic cells) and KLRC2 (activation receptor, expressed on NK cells), cluster 0 did not show any detectable expression. Cluster 1 also lacks expression of KLRC2 while Cluster 2 shows expression of both markers, with higher expression of GNLY. Further analysis revealed that cluster 1 had the lowest expression of perforin (PRF1), granzyme B (GZMB) and interferon-*γ* (IFNG), suggesting that this cluster may include resting or dysfunctional NK cells, with reduced cytotoxic potential. Clusters 0 and 2 display high expression of PRF1, GZMB and IFNG suggesting that they are functionally competent sub-types of NK cells. Cluster 0 is the only NK cluster expressing FCGR3A (Fc-gamma receptor III, also known as CD16), which suggests that it may contain cytotoxic, peripheral blood NK cells (48). Cluster 2 has high expression of ITGAE (CD103) and ZNF683 (HOBIT - regulates immune cell development (49) without any expression of S1PR5 (plays a role in migration of immune cells) and low expression of KLF2 (plays a role in the regulation of NK cell maturation), which suggest that this cluster may include cytotoxic, tissue-resident NK cells (50) (**Figure 8C**). For details about the differential expression markers between the NK clusters refer to **Supplementary Tables 8,**–**10**. We observe varying proportions of NK cell subtypes across our patient cohort, but unfortunately only 9 patients had both scRNAseq and bulk RNAseq, from which only 7 correspond to early stage samples (I, II) (**Figure 8D**), so we could not confidently estimate whether our grouping of bulk RNAseq samples into two patient groups according to NK subtype (indicated by numbers on each barplot) could be associated to the dominance of dysfunctional NK cells in the scRNAseq data.

### Reference-based bulk RNA-seq deconvolution using the scRNAseq from the validation cohort to estimate cell type proportions in our primary cohort reveals the different annotated NK profiles in the LP early stage patients

**Figure 9:**
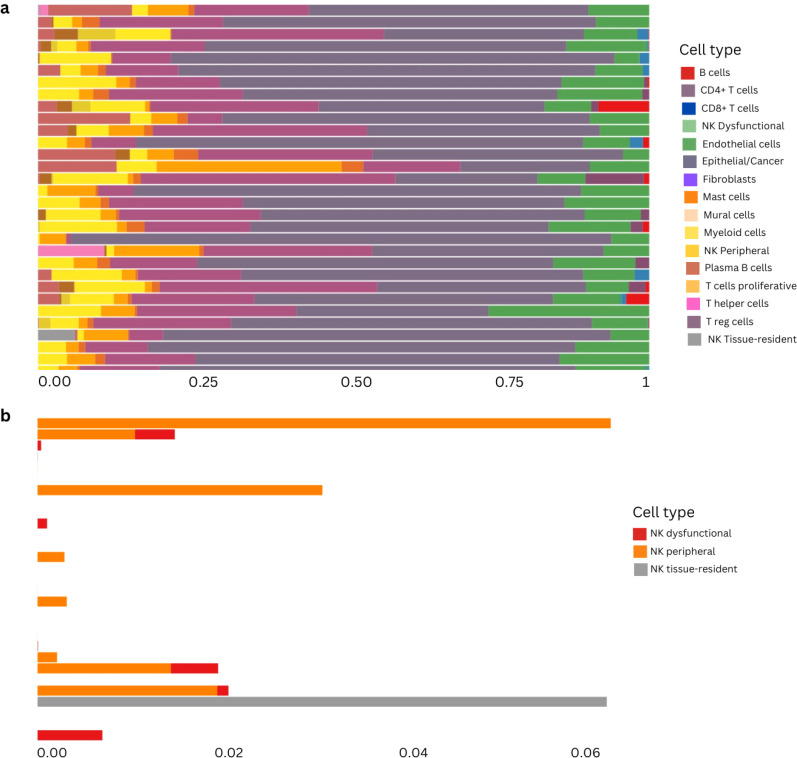
Reference-based deconvolution of primary cohort using BayesPrism method. **(A)** Deconvolution proportions from early stage samples from the LungPredict cohort. NK cells are subdivided into the three subgroups considered above: dysfunctional, peripheral and tissue resident. **(B)** NK subtypes proportions in early stage samples using the cell-type annotations from the scRNAseq object of the validation cohort.

To strengthen and validate our findings regarding cell type composition in the bulk data from our LungPredict cohort, we performed single-cell reference-based bulk RNAseq deconvolution using the scRNAseq data from Vanderbilt as our reference for extracting signatures. We used BayesPrism as implemented in the Omnideconv R package (see Methods) to deconvolve our early stage LungPredict samples. We identified the three different annotated NK subtypes across our samples and found the peripheral cytotoxic NK cell subtype to be the most predominant and the dysfunctional NK subtype to be the least abundant (**Figure 9**).

### Cell type groups are associated with recurrence-free-survival in the validation cohort

**Figure 10:**
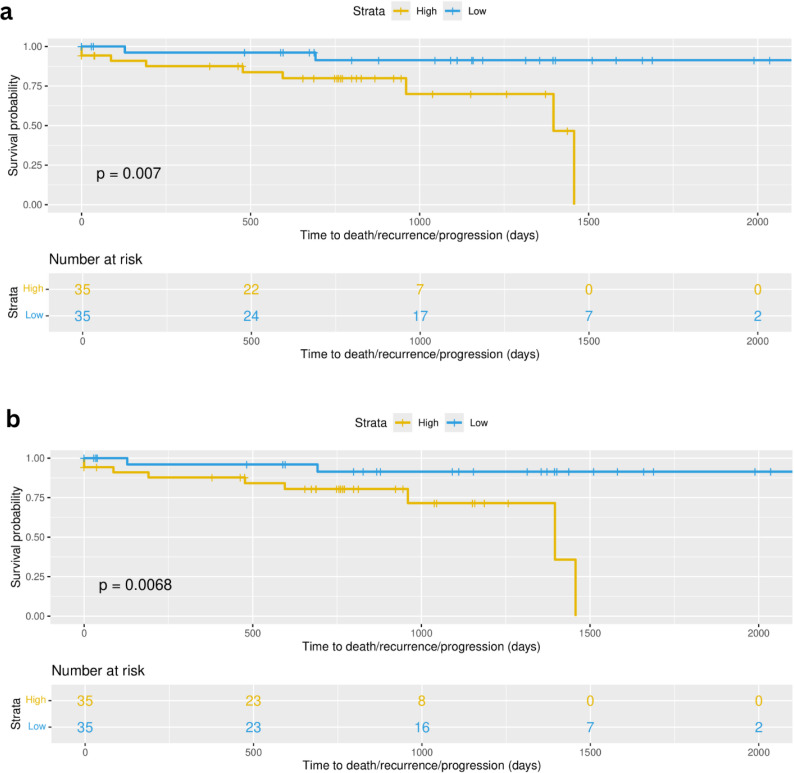
Multivariate cox proportional hazards (Cox PH) models were developed across all selected 10 cell type groups (**Figure 5B**). **(A)** Survival curves based on high and low risk groups using linear predictors after fitting Cox PH model using as covariates cell type groups corresponding to Dendrogram_red_turquoise_black_brown.group_3, Dendrogram_red_turquoise_black_brown.group_9 and Dendrogram_red_turquoise_black_brown.group_combined_1 (p value = 0.007). **(B)** Survival curves based on high and low risk groups using linear predictors after fitting Cox PH model using as covariates cell type groups corresponding to Dendrogram_red_turquoise_black_brown.group_3, Dendrogram_yellow_blue_green.group_2 and Dendrogram_yellow_blue_green.group_3 (p value = 0.0068).

Focusing on early stage disease, we can evaluate the potential association of the immune landscape and disease recurrence. The association of the immune profiles determined through the integration of shared inferred TF activity and the deconvolution features with recurrence was assessed using the mature follow-up available for patients from the validation cohort. CoxPH models were evaluated across all the 10 selected cell type groups and then used to stratify samples based on the linear predictors of the model. Kaplan Meier analysis and log rank tests were used to assess the difference between risk groups (see Methods). Two multivariate models were found as significant after log rank test (p value = 0.007 and p value = 0.0068) (**Figure 10**). In model 1, the variables (covariates) that are most associated to recurrence free survival were Dendrogram_red_turquoise_black_brown.group_3, including resting NK cells, Dendrogram_red_turquoise_black_brown.group_9 and Dendrogram_red_turquoise_black_brown.group_combined_1, including more active/cytotoxic NK cells with other immune cells like neutrophils, T cells and activated dendritic cells.

Model 2 also contains as covariate the Dendrogram_red_turquoise_black_brown.group_3 feature, and additionally two other cell type groups: Dendrogram_yellow_blue_green.group_2, containing the NK resting subgroup as well as other resting immune cells (CD4, dendritic, Mast), and Dendrogram_yellow_blue_green.group_3, containing the more active NK subgroup in combination with T cells (CD4 and CD8) and dendritic cells in their active state (see **Supplementary Table 7** for detailed composition of the cell groups). This result is limited by small sample size (n=70) and a low event rate (n=11), however the results serve as preliminary evidence for the applicability of transcriptomically defined immune patient profiles in real world outcomes among early stage lung adenocarcinoma patients.

### TCGA LUAD cohort analysis confirms similar immune infiltration profiles across early stage patients

**Figure 11:**
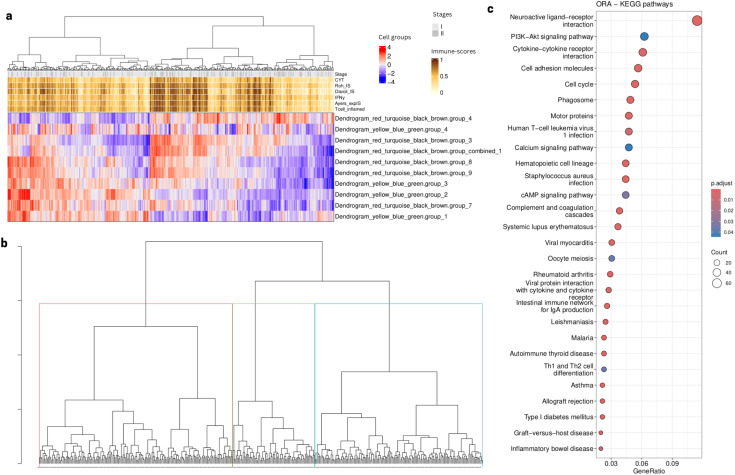
TCGA analysis using selected cell type groups from **Figure 9A**. **(A)** Heatmap showing cell type groups scores after projection using the computed deconvolution and the inferred TF activity. **(B)**Samples dendrogram using hierarchical clustering based on the cell type groups scores **(C)**. Dotplot showing KEGG pathways (p value < 0.05) related to the enrichment of DEG (padj < 0.05, abs(log_2_FoldChange) > 1) after comparison between patient Cluster 1 and 3 (red and blue in panel **B**, respectively).

To further test the validity of our findings, we selected the 399 early stage (I,II) lung adenocarcinoma (LUAD) from TCGA. We performed immune cell type deconvolution and inferred TF activity across these samples as described above. We then projected and recreated the 10 selected cell type groups (see above) using the same TF modules found in the analysis mentioned above using early stage samples in the primary and in the validation cohorts. Our results showed three patient clusters related to distinct immune infiltration profiles. Two of the three patient clusters revealed similar expression patterns as the ones found in the LP and Vanderbilt cohorts (**Figure 11A**) and we identified patient clusters 1 (red) and 3 (green) as the clusters defined by two opposite NK profiles (**Figure 11B**). We then performed a differential expression analysis and a functional enrichment analysis using the KEGG database, identifying 1518 differentially expressed genes (padj <.00001, abs(log_2_FoldChange) > 1.5) revealing an enrichment in immunological and cytotoxic related pathways (p value < 0.05) (**Figure 11C**).

### Survival analysis in TCGA revealed that both resting and activated NK subtypes are significant predictors of survival

**Figure 12:**
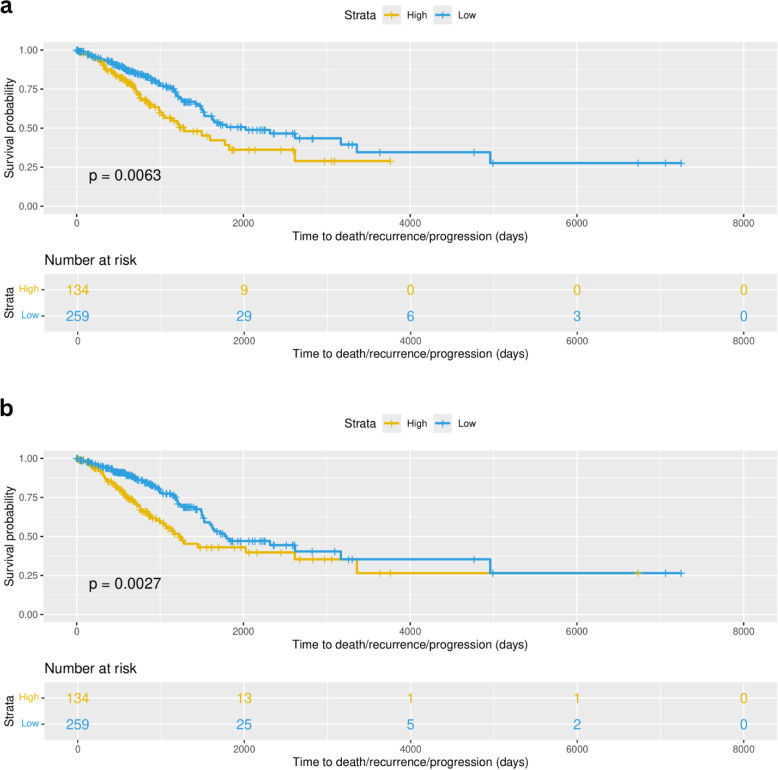
Survival curves corresponding to the analysis done for TCGA-LUAD (393 early stage patients). **(A)** Survival curves showed a significant difference (p value = 0.0063) of survival using formula 1 (Surv(time, status) ~ dendrogram_red_turquoise_black_brown.group_3) when comparing high-risk patients (yellow) and low-risk (blue) patients defined based on the risk scores. **(B)** Survival curves showed a significant difference (p value = 0.0027) of survival using formula 2 (Surv(time, status) ~ dendrogram_red_turquoise_black_brown.group_4) when comparing high-risk patients (yellow) and low-risk (blue) patients defined based on the risk scores.

Linear predictors from univariate cox proportional hazards (coxPH) models across all the 10 selected cell groups were evaluated to stratify patients based on their risk-scores, subsequently computing the survival curves through Kaplan Meier analysis and testing whether the survival between the two groups is significantly different (p value < 0.01). In this dataset we applied stricter filtering due to the high number of patients (n=393), stratifying as high-risk only the top 34% of patients (based on their risk scores) and the remaining 66% as “low-risk”. Two models were found to be significantly associated with the survival time of patients (**Figure 12**). Cell type groups dendrogram_red_turquoise_black_brown.group_3 and dendrogram_red_turquoise_black_brown.group_4 with p value = 0.0063 and p value = 0.0027, respectively. The first cell type group corresponds to the subgroup of resting NK cells with macrophages M1 and the second one corresponds to the NK subgroup in combination with cancer, fibroblasts, dendritic, and Thelper cells (see **Supplementary Table 7**). Both these features were predictors of survival in the univariate models. These results suggest an important association between these NK subtypes and patient survival.

### Patient subgroups identified are related to oncogene and tumor suppressor TF modules

**Figure 13:**
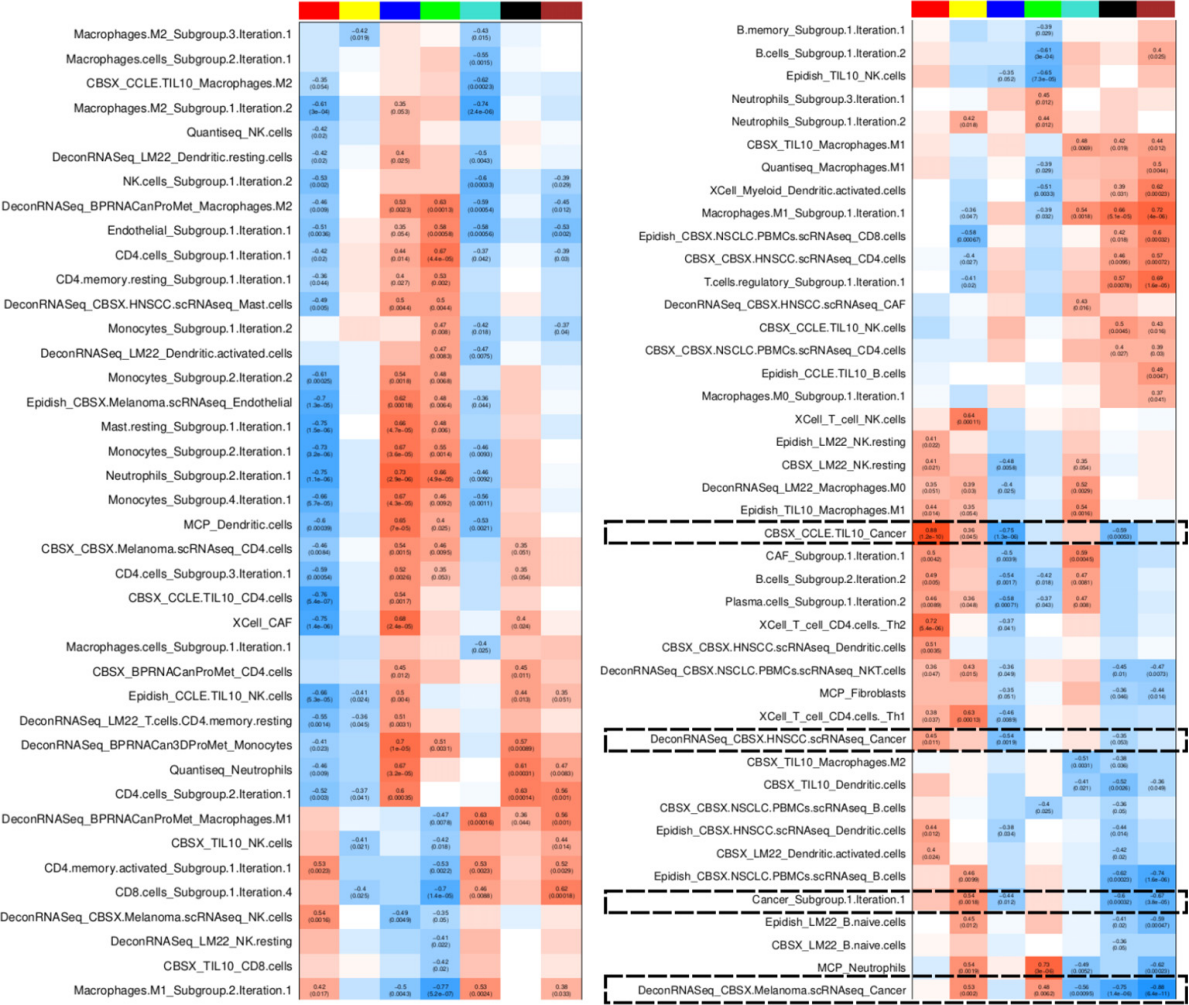
TF module characterisation based on association with grouped deconvolution features in early stage Lung Predict samples. The heatmap shows Pearson correlation between TF module scores and deconvolution features, highlighting cancer-related features. Colors represent levels of correlation (darker red implies high positive correlation, darker blue implies high negative correlation). Statistics are shown only for significantly correlated pairs (p value < 0.05).

To further investigate the functional mechanisms leading to the subgrouping of patients into 2 categories according to their TME landscapes, we further explored the association between TF modules and deconvolution features. In particular we highlight the modules that are associated with abundance of cancer cells as potentially capturing oncogenic processes while other modules negatively correlated with cancer cells could be considered as tumor suppressor processes (**Figure 13**). The module that is more strongly positively correlated with cancer cell estimates is red, which shows strong repression of Trail and p53 pathways and activation of MAPK, VEGF and Hypoxia and is strongly positively correlated to the presence of resting NK cells and negatively to the presence of active NK cells. The black and brown modules are negatively correlated with the same features and show instead strong activation of immune processes (NFkB and TFGb). The repression of module red clearly sets patients in cluster 1 apart (c.f. **Figure 5C**). The TF activity profiles across early stage Lung Predict samples of TFs contained in each module are shown in **Supplementary Figure 8.**

Since TF activities are estimated based on bulk RNAseq, we cannot be sure of whether these pathways are activated mainly in the cancer cells or the correlation directly reflects the tumor sample purity. However, combining these two types of features we have demonstrated that discordance between deconvolution signatures might simply reflect substantial differences in the subtypes of cells they refer to.

## Discussion

This study leveraged integrative computational approaches to dissect immune heterogeneity in the tumor microenvironment of lung adenocarcinomas. Integrating bulk transcriptomics with bioinformatic analyses for cell type deconvolution and TF activity inference, we identified profiles associated with dual immune cell phenotypes (51).

Specifically, our combined analysis suggested the presence of two subgroups of natural killer (NK) cells. One subgroup is associated with a high proportion of cancer cells and CAFs and could be potentially associated with a “resting” or “dysfunctional” behavior. Dysfunctional NK cells are characterized by reduced proliferation and cytotoxic capabilities. In contrast, we inferred a high presence of B-cells, T-cells and NK cells in early stage samples with high immune-scores. This different group of NK cells may display cytotoxic capabilities and might even be subdivided into two NK profiles, depending on co-occurrence of other cell types, namely endothelial cells. Focusing on early stage (stage I and II) patient samples, we confirmed these dual NK subgroups in an independent LUAD cohort and in the 399 stage I and II LUAD samples from TCGA after further characterizing them in an scRNAseq dataset. Interestingly, in the scRNAseq data analysis, we identified three major NK clusters. We characterized these three clusters as resting/dysfunctional, circulating cytotoxic and tissue-resident cytotoxic NK cells. The single-cell analysis provided independent validation of the computationally defined NK cell subtypes/states, and provided further resolution into tissue-resident versus circulating NK cell subsets. Finally, we were able to show that our engineered features based on cell type groups, which take into account TF activity profiles to estimate presence of groups of different cell types, have predictive value on recurrence free survival (in our validation cohort) and on overall survival (in the TCGA cohort).

To summarize, we revealed a striking duality in NK cell phenotypes across three independent cohorts, with NK subsets displaying signatures of dysfunctional exhaustion versus cytotoxic competence. Dysfunctional NK cells have reduced proliferative and functional capacity, resulting from constant exposure to immune suppressive signals in the tumor microenvironment. Our findings align with other recent studies showing phenotypic heterogeneity in NK cells and other immune cell types in the context of cancer (52, 53) and with reports that NK cell states might be essential for response to PD-1/PD-L1 blockers (54) and key players in immunotherapy (55, 56). Beyond those results, our approach is a first step towards delineating the type of inter-cellular interactions that could be established in the TME in connection to the presence of these two NK cell subtypes.

Overall, our study sheds light on the significant diversity of immune cells in the lung cancer microenvironment. The integrated computational frameworks provide an accessible, robust and general methodology for immune profiling of tumor samples via bulk RNAseq.

Immune cell dysfunction arises from continuous stimulation in a persistent inflammatory environment. In the tumor microenvironment (TME), the presence of various immune suppressive signals exacerbates immune cell dysfunction leading to tumor progression and metastasis (57). The ability to resolve immune cell dysfunction versus activation states could significantly improve prognostic models and prediction of immunotherapy response (58). Whether these dysfunctional characteristics are a result of exhaustion or senescence will need to be determined (59). Our approach is a very step towards delineating the type of inter-cellular interactions that could be established in the TME in connection to the presence of these two NK cell subtypes.

Our exploration of the single cell data further strengthens the hypothesis that there are two major subgroups of NK cells, dysfunctional/resting and functional, associated with immune cells presence and that patients might be characterized based on the dominance of either of these two NK cell subgroups. It could be speculated that the profile of NK cell subtypes present could be related to response to immune checkpoint blockers. However, early stage LUAD patients are still rarely treated with this type of therapy, while only a few patients in Lung Predict received it, requiring alternative cohorts to validate this hypothesis. However, we note that in any non-pharmacologically treated tumor a strong immune response is likely to improve survival, potentially explaining why the active NK subtype, which associates with M1-like macrophages, could also improve survival in cases that are treated by surgery alone, as those included in our primary and validation cohorts.

We note that our initial analysis on the Lung Predict cohort across stages suggests that the duality in NK cells populations is not limited to early stage disease. Looking forward, extension of these analyses across lung cancer stages and histological subtypes could provide valuable insights into reprogramming of the immune microenvironment during progression. Incorporating spatial and proteomic data could help further resolve the tissue localization and functional capacities of distinct immune cell subsets in lung tumors. Ultimately, comprehensive mapping of immune heterogeneity in lung cancer provides a path towards more precise immunotherapeutic strategies (53, 60).

Nevertheless, this study has several limitations to be considered. First, the sample size was relatively small, with only 62 lung adenocarcinomas in the primary analysis cohort and 70 in the validation cohort. The number of samples included in our analysis from TCGA is considerable (399) and helped us confirm our findings, but the cohort is likely to be less homogeneous. Larger studies on deeply clinically characterized samples will be needed to further validate the findings. Second, we utilized only transcriptomic data, which provides an incomplete picture of cellular states compared to integrating proteomics and adding spatial resolution. Third, our study lacked longitudinal samples, with which we could assess how immune profiles change over time and with therapy. Fourth, bulk transcriptomics may underestimate certain rare cell populations that are better captured by single-cell sequencing. Our in-depth analysis of 15 samples for which scRNAseq was available and using NK populations identified therein helped us confirm the presence of the NK subtypes in our bulk RNAseq datasets. Fifth, the specific deconvolution algorithms used can impact results, and incorporating additional methods could provide further validation. Finally, functional validations to directly test immune cell cytotoxicity or dysfunctional profiles in NK cells were not performed. This would require either *in-vitro* experiments or very deep characterisation of clinical samples that are beyond the scope of this study.

Overall, this proof-of-concept study demonstrated the potential of integrated computational immunology techniques to identify signatures of immune cell dysfunction from bulk tumor profiling. However, further experimental and clinical validations are needed to fully characterize the phenotypic diversity of anti-tumor immune responses in lung adenocarcinoma patients.

## Conclusion

In summary, our multi-omics computational framework elucidated heterogeneous immune microenvironments in lung adenocarcinoma. Deconvolution and TF activity analysis identified groups of immune cells with coordinated regulation/states. The ability to resolve dysfunctional/resting versus activated immune cell states from bulk tumor profiling could have important implications for prognosis and prediction of response to immunotherapy, as suggested by our preliminary evidence of an association to survival in 3 early LUAD cohorts. Further characterization of dynamic immune reprogramming during cancer progression and therapy response represents an important future direction. We make the RNAseq datasets from our Lung Predict cohort and all the code available to the research community, hoping to contribute to reproducibility and open-research practices for the ultimate benefit of patients.

## Data Availability

The primary LUAD cohort (Lung Predict) transcriptomics data is available on NCBI GEO with study number GSE251840. The validation LUAD cohort (Vanderbilt) data is available on Zenodo under accession number 7878082. The code to reproduce the analysis and figures is available on github at https://github.com/VeraPancaldiLab/LungPredict1_paper.

## Supplementary material

Supplementary Figure 1 TFs modules characterization from early stage Lung predict samples. **(A)** Number of TFs across each of the 7 modules. **(B)** Module association between TFs modules scores and pathway values (only showing significant correlations considering p value < 0.05). **(C)** Heatmap of the TF activity of the 20 hub TFs across samples, showing their related module as the color annotation on the right. **(D)** Reactome enrichment results from unique target genes from hub TFs of each module.

Supplementary Figure 2 TFs modules classification and characterization from analysis on early stage samples from Lung Predict cohort. **(A)** Construction of weighted TFs modules based on inferred co-activity. **(B)** Hierarchical clustering based on association values between TFs modules and pathway activities. **(C)** Biplot representing the contribution of the top 6 pathways classifying the TFs modules. **(D)** Contribution of each pathway on the TFs module classification.

Supplementary Figure 3 Analysis of combined LungPredict and Vanderbilt validation cohort A. **(A)** difference between the LP and Vanderbilt cohorts on normalized counts was evident and treated as a batch effect **(B)** Heatmap showing the Pearson correlation between the principal components and the metadata variables (the darker the green the higher the correlation). p values 0, 0.0001, 0.001, 0.01, 0.05, 1 correspond to ‘****’, ‘***’, ‘**’, ‘*’, ‘‘ respectively. **(C)** PCA plot using TFs activity values after calculating it independently in each cohort, shows the difference between cohorts was removed. **(D)** Heatmap showing no significant correlation between cohorts (treated here as batches) and the principal components (PCs) using TFs activity.

Supplementary Figure 4 PCA analysis to assess batch effect within the validation cohort. **(A)** PCA of validation cohort (Vanderbilt) normalized counts before batch correction **(B)**. PCA of validation cohort normalized counts after batch effect removal by Combat_seq from the sva R package (61 (v3.50.0)) to maintain the integrity of the raw counts.

Supplementary Figure 5 KEGG pathway diagram of differentially expressed genes between two patient clusters identified in the Vanderbilt cohort early stage samples (c.f. **Figure 9A**). The diagram shows the “Natural Killer Cell mediated cytotoxicity pathway” produced using the pathview R package (62 (v1.42.0)) components and interactions, highlighting downregulation of inhibitory (KIR3DL1/2) receptors as well as protein kinase C (PKC).

Supplementary Figure 6 UMAP of scRNAseq data from 15 Vanderbilt cohort patients (31). UMAP shows no batch effect influence in the cell based clustering.

Supplementary Figure 7 Automatic cluster annotation from Vanderbilt scRNA cohort using reference expression datasets with curated cell type labels. **(A)** Cluster automation using Human Primary Cell Atlas. **(B)** Cluster annotation using Database Immune Cell Expression Data. **(C)** Cluster annotation using Monaco database. **(D)** Cluster annotation using Blueprint Encode Data.

Supplementary Figure 8 TFs activity of module composition from TF modules. Modules black, red, blue, brown, green, turquoise and yellow correspond to Figures **(A–G)** respectively.

Supplementary Text 1 related to **Supplementary Figure 3**, **Supplementary Figure 4** Evaluation of batch effects within and between cohorts: To assess comparability between the Lung Predict and Vanderbilt early stage cohorts, we performed a PCA analysis using the R package PCAtools (63 (v2.14.0)) where we joined the two datasets and tested whether they separated or not. As expected, there is a big difference between the two cohorts based on normalized counts (**Supplementary Figure 3A**) with a pearson correlation of 1 (p value < 0.0001) between cohort (here batch) and the first principal component (**Supplementary Figure 3B**). Instead of removing the batch effect, which potentially can also eliminate some important biological differences, and since our analysis does not directly use normalized counts, we decided to calculate TFs activity independently for each cohort and assess again for batch effects. As expected, calculation of the inferred TFs activity removed the batch effects between the two cohorts (**Supplementary Figure 3C**) showing no correlation (r = 0.01) between the cohorts and the PC1 (**Supplementary Figure 3D**). Once we confirmed that the two datasets can be comparable when looking at the TF activity profiles, we performed the previously described analysis only on the validation cohort to assess for within-dataset batch effects. A PCA analysis identified two main groups confounded by batches (**Supplementary Figure 4A**). For this reason, we performed both our TFs inference analysis and immune cell type deconvolution calculation independently for each batch. We then concatenated our results and saw that even though the TFs analysis was not affected by the batch effect, this was still present in the deconvolution results. We then used Combat_seq from the sva R package (61 (v3.50.0)) to remove batch effects from our counts and maintain the integrity of the raw counts (**Supplementary Figure 4B**). Finally, after log_2_(TPM + 1) normalization we calculated deconvolution features from batch corrected datasets.

Supplementary Table 1 Deconvolution methods and signatures.

Supplementary Table 2 Immune-scores hallmarks.

Supplementary Table 3 Composition of deconvolution features subgroups on all samples from Lung Predict cohort.

Supplementary Table 4 Composition of TF modules obtained from all samples from Lung Predict cohort.

Supplementary Table 5 Composition of deconvolution features subgroups on early stage samples from Lung Predict cohort.

Supplementary Table 6 Composition of TF modules obtained from early stage samples from Lung Predict cohort.

Supplementary Table 7 Composition of cell groups obtained from early stage samples from Lung Predict cohort.

Supplementary Table 8 Differential expression markers between NK peripheral (pct.1) and NK dysfunctional (pct.2) (p_val_adj <0.05 and abs(avg_log2FC) > 1).

Supplementary Table 9 Differential expression markers between NK peripheral (pct.1) and NK Tissue resident (pct.2) (p_val_adj <0.05 and abs(avg_log2FC) > 1).

Supplementary Table 10 Differential expression markers between NK Dysfunctional (pct.1) and NK Tissue resident (pct.2) (p_val_adj <0.05 and abs(avg_log2FC) > 1).
