# Supplementary material for: Transcriptomics profiling of the non-small cell lung cancer microenvironment across disease stages reveals dual immune cell-type behaviors
Source: Front Immunol. 2024 Oct 31;15:1394965. doi: 10.3389/fimmu.2024.1394965 (PMC11600981; doi:10.3389/fimmu.2024.1394965)
Supplement: Supplementary Figure 1 — TFs modules characterization from early stage Lung predict samples. (A) Number of TFs across each of the 7 modules. (B) Module association between TFs modules scores and pathway values (only showing significant correlations considering p value < 0.05). (C) Heatmap of the TF activity of the 20 hub TFs across samples, showing their related module as the color annotation on the right. (D) Reactome enrichment results from unique target genes from hub TFs of each module. [file Presentation1.pdf]

Supplementary Materials

Transcriptomics Profiling of the Non-Small Cell Lung Cancer (NSCLC) Microenvironment Across Disease Stages Reveals Dual Immune Cell-Type Behaviors

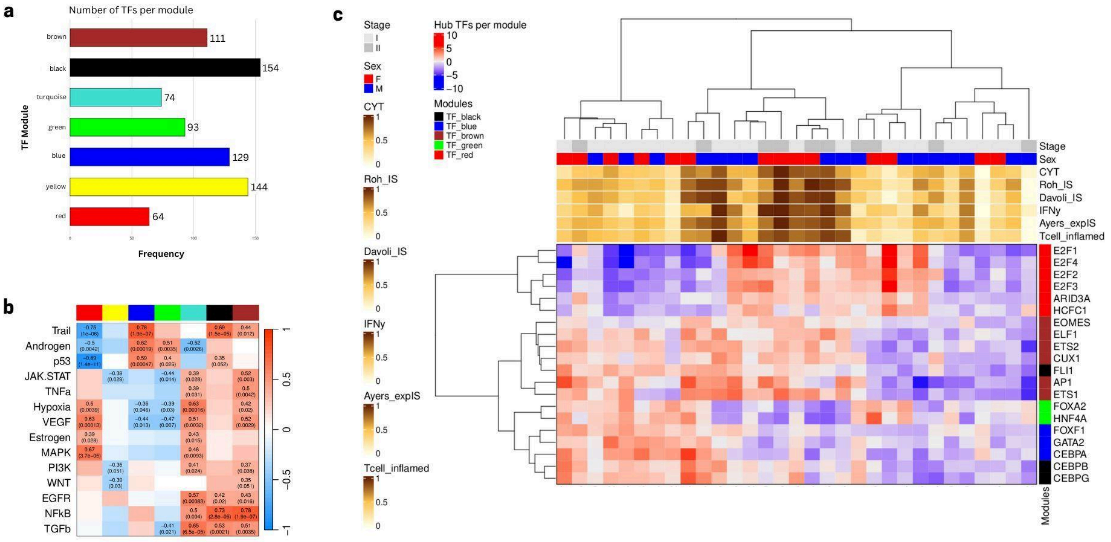

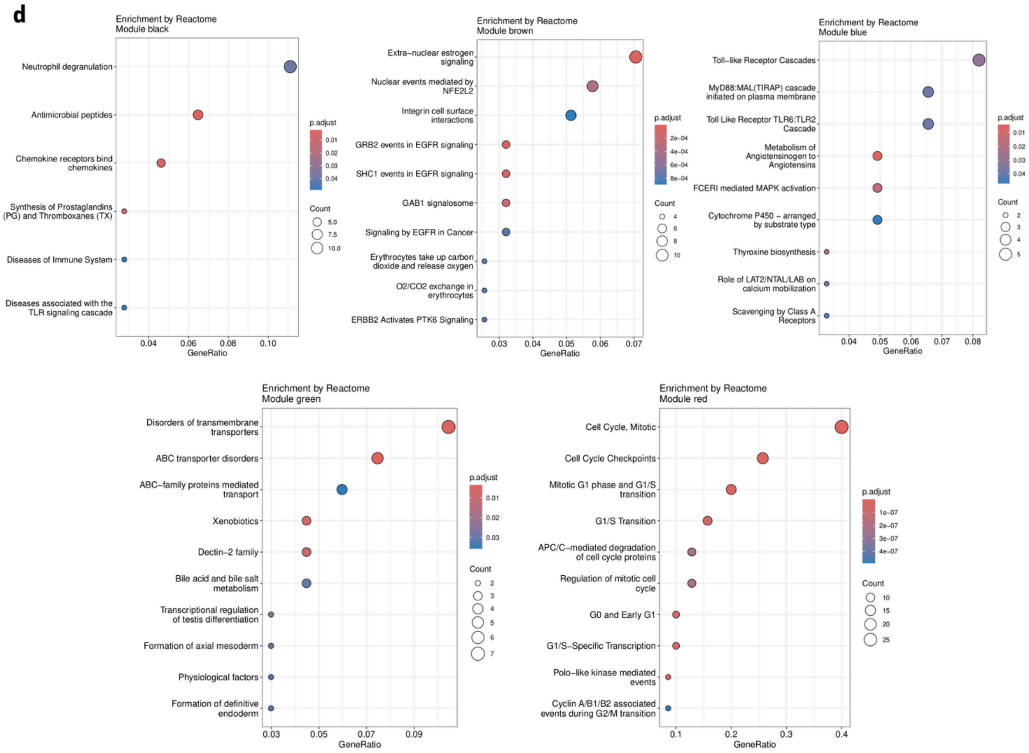

Supplementary Figure 1. TFs modules characterization from early stage Lung predict samples. A. *Number of TFs across each of the 7 modules.* ***B.*** *Module association between TFs modules scores and pathway values (only showing significant correlations considering p value < 0.05).* ***C.*** *Heatmap of the TF activity of the 20 hub TFs across samples, showing their related module as the color annotation on the right.* ***D.*** *Reactome enrichment results from unique target genes from hub TFs of each module.*

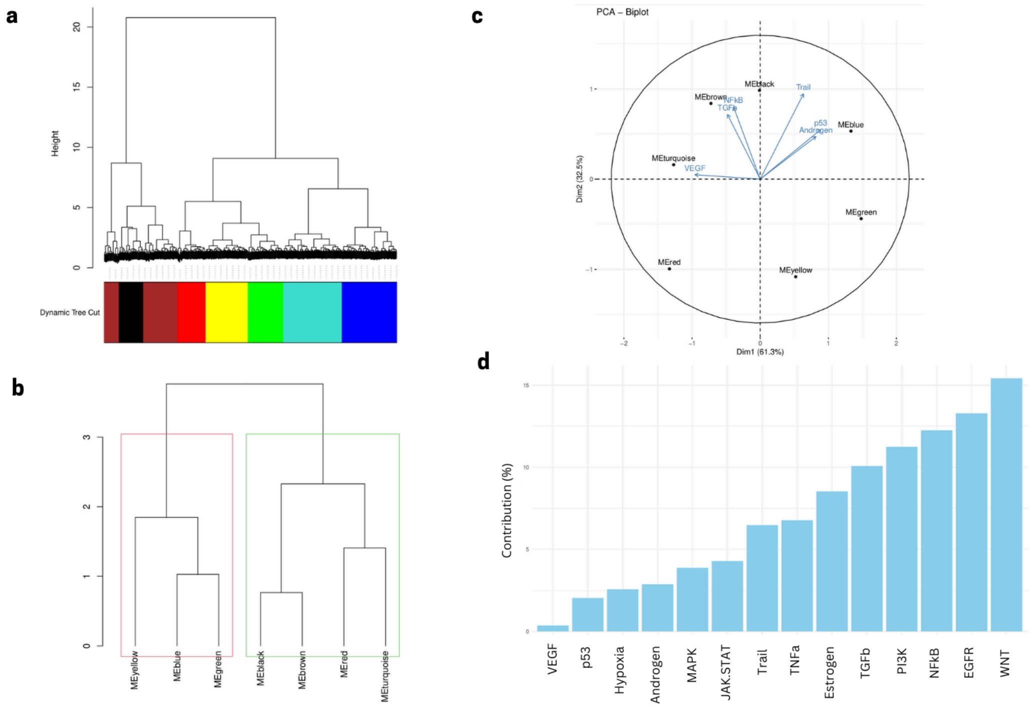

Supplementary Figure 2. TFs modules classification and characterization from analysis on early stage samples from Lung Predict cohort. A. *Construction of weighted TFs modules based on inferred co-activity.* ***B.*** *Hierarchical clustering based on association values between TFs modules and pathway activities.* ***C.*** *Biplot representing the contribution of the top 6 pathways classifying the TFs modules.* ***D.*** *Contribution of each pathway on the TFs module classification.*

Supplementary Text 1 related to Supplementary Figure 3 and 4

Evaluation of batch effects within and between cohorts:

To assess comparability between the Lung Predict and Vanderbilt early stage cohorts, we performed a PCA analysis using the R package PCAtools (Blighe et al. 2024 (v2.14.0)) where we joined the two datasets and tested whether they separated or not. As expected, there is a big difference between the two cohorts based on normalized counts (**Supplementary Figure 3A**) with a pearson correlation of 1 (p value < 0.0001) between cohort (here batch) and the first principal component (**Supplementary Figure 3B**). Instead of removing the batch effect, which potentially can also eliminate some important biological differences, and since our analysis does not directly use normalized counts, we decided to calculate TFs activity independently for each

cohort and assess again for batch effects. As expected, calculation of the inferred TFs activity removed the batch effects between the two cohorts (**Supplementary Figure 3C**) showing no correlation (r = 0.01) between the cohorts and the PC1 (**Supplementary Figure 3D**). Once we confirmed that the two datasets can be comparable when looking at the TF activity profiles, we performed the previously described analysis only on the validation cohort to assess for within-dataset batch effects. A PCA analysis identified two main groups confounded by batches (**Supplementary Figure 4A**). For this reason, we performed both our TFs inference analysis and immune cell type deconvolution calculation independently for each batch. We then concatenated our results and saw that even though the TFs analysis was not affected by the batch effect, this was still present in the deconvolution results. We then used Combat_seq from the sva R package (Leek et al. 2024 (v3.50.0)) to remove batch effects from our counts and maintain the integrity of the raw counts (**Supplementary Figure 4B**). Finally, after $\log_2(\mathrm{TPM} + 1)$ normalization we calculated deconvolution features from batch corrected datasets.

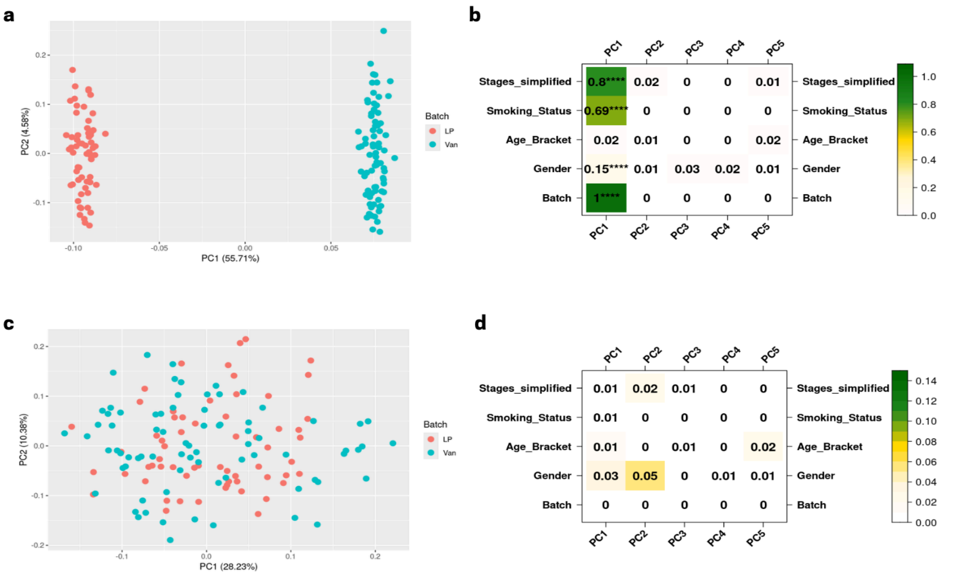

Supplementary Figure 3. Analysis of combined LungPredict and Vanderbilt validation cohort ***A. A difference*** *between the LP and Vanderbilt cohorts on normalized counts was evident and treated as a batch effect* ***B.*** *Heatmap showing the Pearson correlation between the principal components and the metadata variables (the darker the green the higher the correlation). p values 0, 0.0001, 0.001, 0.01, 0.05, 1 correspond to '****', '***', '**', '*', '' respectively.* ***C.*** *PCA*

plot using TFs activity values after calculating it independently in each cohort, shows the difference between cohorts was removed. ***D.*** *Heatmap showing no significant correlation between cohorts (treated here as batches) and the principal components (PCs) using TFs activity.*

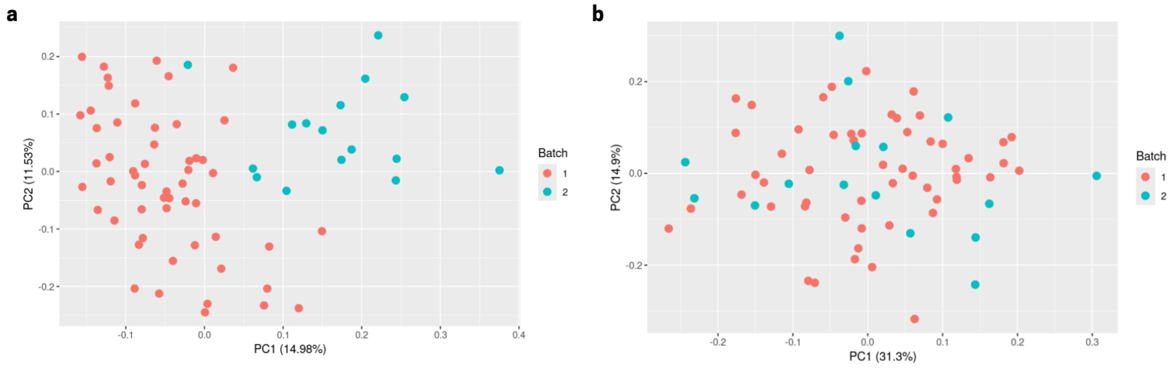

Supplementary Figure 4. PCA analysis to assess batch effect within the validation cohort. A. *PCA of validation cohort (Vanderbilt) normalized counts before batch correction* ***B.*** *PCA of validation cohort normalized counts after batch effect removal by Combat_seq from the sva R package (Leek et al. 2024 (v3.50.0)) to maintain the integrity of the raw counts.*

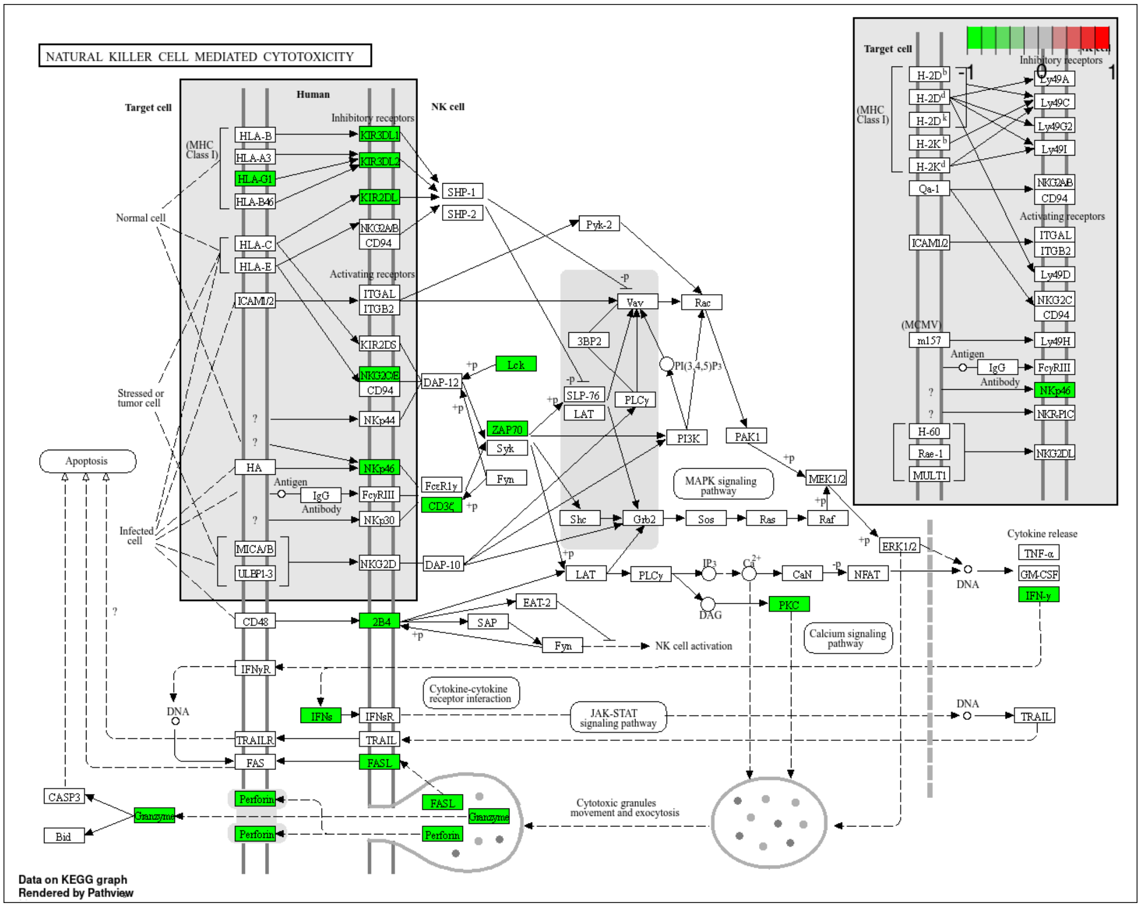

Supplementary Figure 5*. *KEGG pathway diagram of differentially expressed genes between two patient clusters identified in the Vanderbilt cohort early stage samples *(c.f.* ***Figure 9A****). The diagram shows the "Natural Killer Cell mediated cytotoxicity pathway" produced using the pathview R package (Luo et al. 2013 (v1.42.0)) components and interactions, highlighting downregulation of inhibitory (KIR3DL1/2) receptors as well as protein kinase C (PKC).*

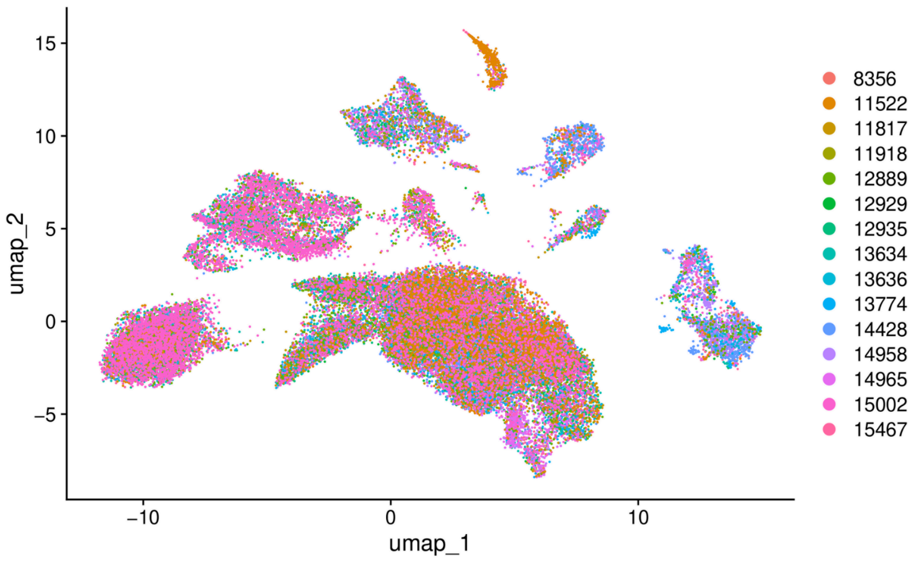

***Supplementary Figure 6**. **UMAP of scRNAseq data from 15 Vanderbilt cohort patients (Senosain et al. 2023)**. UMAP shows no batch effect influence in the cell based clustering.*

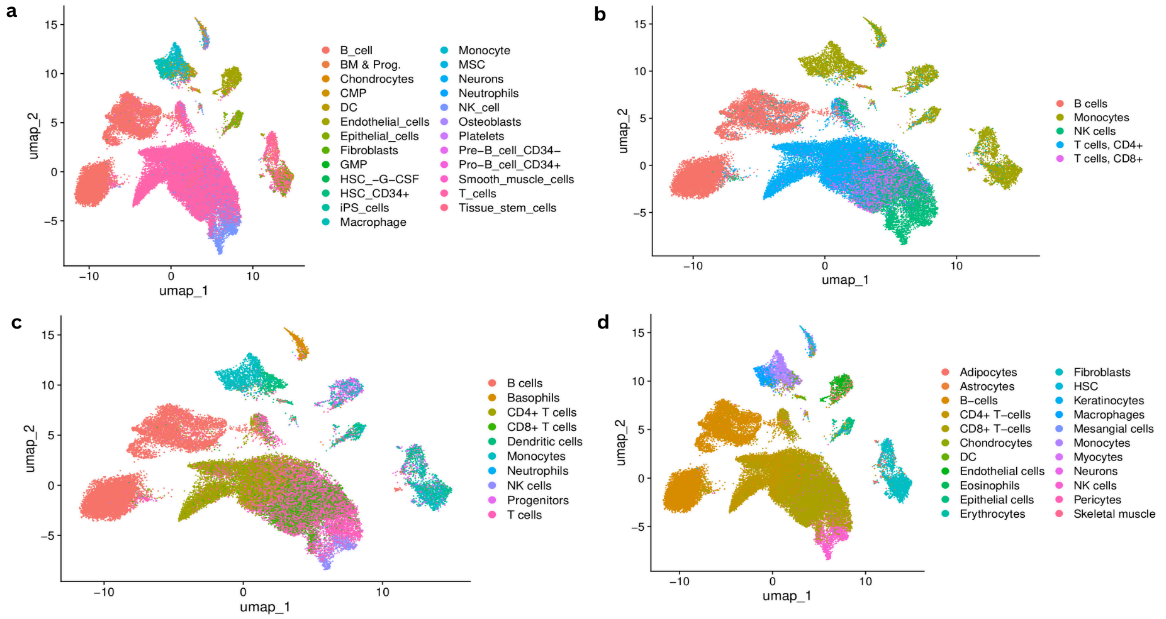

***Supplementary Figure 7**. **Automatic cluster annotation from Vanderbilt scRNA cohort using reference expression datasets with curated cell type labels. A.** Cluster automation using Human Primary Cell Atlas. **B.** Cluster annotation using Database Immune Cell Expression Data. **C.** Cluster annotation using Monaco database. **D.** Cluster annotation using Blueprint Encode Data.*

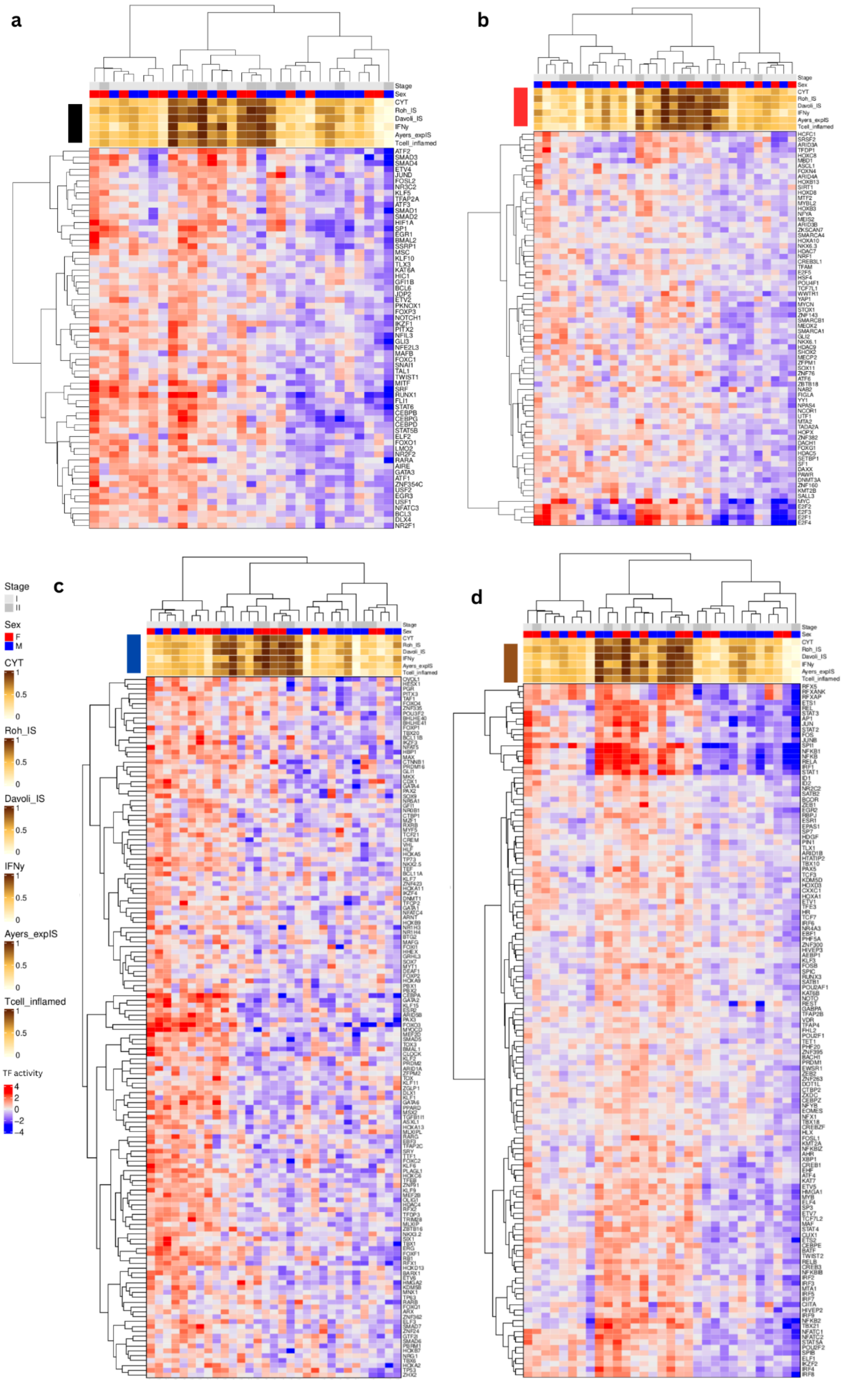
a
b
c
d
Stage
Sex
CYT
Roh_IS
Davoli_IS
IFNy
Ayers_expIS
Tcell_inflamed
Stage
I
II
Sex
F
M
CYT
Roh_IS
Davoli_IS
IFNy
Ayers_expIS
Tcell_inflamed
TF activity

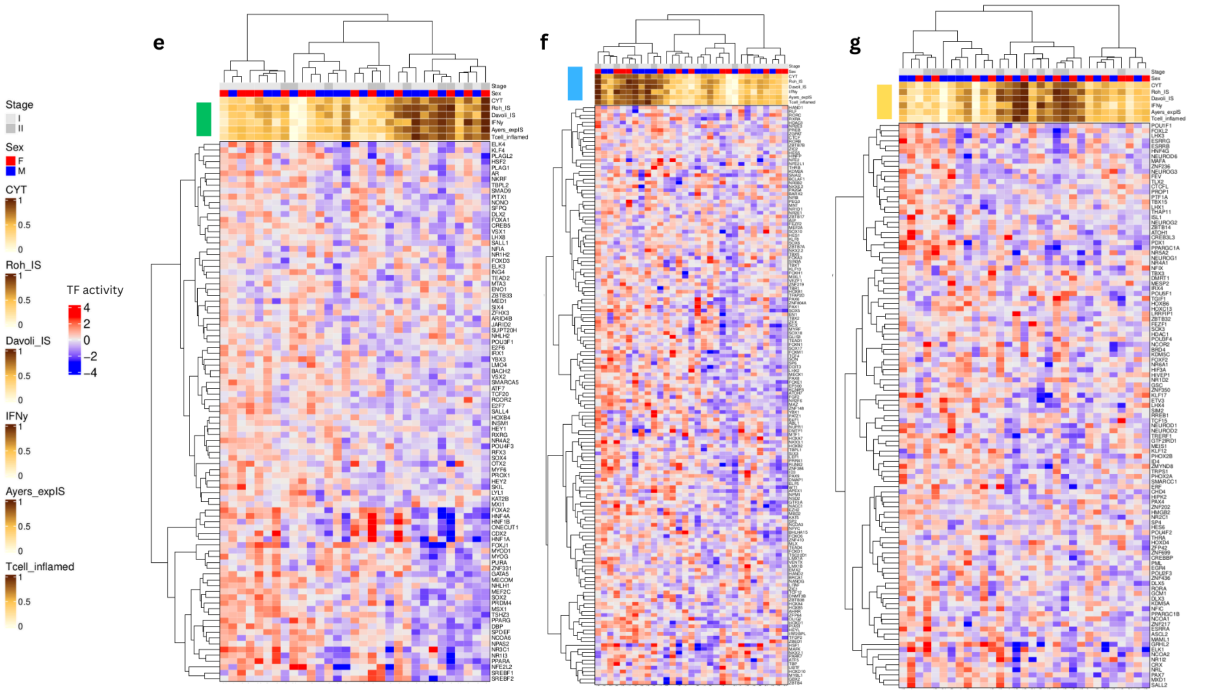

Supplementary Figure 8. TFs activity of module composition from TF modules. Modules black, red, blue, brown, green, turquoise and yellow correspond to Figures A, B, C, D, E, F, G respectively.

Supplementary Tables

Supplementary Table 1: Deconvolution methods and signatures.

Deconvolution methods	Reference
CIBERSORTx	Newman et al. (2019)
quanTIseq	Finotello et al. (2019)
DeconRNAseq	Gong et al. (2013)
EpiDISH	Teschendorff et al. (2017)
MCP	Becht et al. (2016)
XCell	Aran et al. (2017)

Deconvolution signatures	Reference
LM22	Newman et al. (2019)
CBSX.NSCLC.PBMCs.scRNAseq	Xie et al. (2023)
CBSX.Melanoma.scRNAseq	Xie et al. (2023)
CBSX.HNSCC.scRNAseq	Xie et al. (2023)
CCLE_TIL10	Xie et al. (2023)
TIL10	Finotello et al. (2019)
BPRNACan	Xie et al. (2023)
BPRNACanProMet	Xie et al. (2023)
BPRNACan3DProMet	Xie et al. (2023)

Supplementary Table 2: Immune-scores hallmarks

Immune-score	Reference
Cytolytic activity (CYT)	Rooney et al. (2015)
Roh immune score (Roh_IS)	Roh et al. (2017)
Chemokine signature (chemokines)	Messina et al. (2012)
Davoli immune signature (Davoli_IS)	Davoli et al. (2017)
IFNy signature (IFNy)	Ayers et al. (2017)

Expanded immune signature (Ayers_expIS)	Ayers et al. (2017)
T-cell inflamed signature (Tcell_inflamed)	Ayers et al. (2017)
Repressed immune resistance (RIR)	Jerby-Arnon et al. (2018)
Tertiary lymphoid structures signature (TLS)	Cabrita et al. (2020)

Supplementary Table 3: Composition of deconvolution features subgroups on all samples from Lung Predict cohort.

Cell subgroups	Methods-signatures
**B.cells_Subgroup.3.Iteration.1**	DeconRNASeq_CBSX.NSCLC.PBMCs.scRNAseq_B.cells<br>CBSX_CBSX.NSCLC.PBMCs.scRNAseq_B.cells
**B.cells_Subgroup.1.Iteration.2**	DeconRNASeq_CBSX.HNSCC.scRNAseq_B.cells<br>DeconRNASeq_CBSX.Melanoma.scRNAseq_B.cells
**B.cells_Subgroup.2.Iteration.2**	CBSX_CBSX.Melanoma.scRNAseq_B.cells<br>B.cells_Subgroup.1.Iteration.1
**B.memory_Subgroup.1.Iteration.1**	DeconRNASeq_LM22_B.memory.cells<br>Epidish_LM22_B.memory.cells
**Macrophages.cells_Subgroup.1.Iteration.1**	CBSX_CBSX.HNSCC.scRNAseq_Macrophages.cells<br>DeconRNASeq_CBSX.Melanoma.scRNAseq_Macrophages.cells
**Macrophages.cells_Subgroup.1.Iteration.2**	Epidish_CBSX.Melanoma.scRNAseq_Macrophages.cells<br>Macrophages.cells_Subgroup.1.Iteration.1
**Macrophages.cells_Subgroup.1.Iteration.3**	CBSX_CBSX.Melanoma.scRNAseq_Macrophages.cells<br>Macrophages.cells_Subgroup.1.Iteration.2
**Macrophages.M0_Subgroup.1.Iteration.1**	Epidish_LM22_Macrophages.M0<br>CBSX_LM22_Macrophages.M0
**Macrophages.M1_Subgroup.1.Iteration.1**	DeconRNASeq_CCLE.TIL10_Macrophages.M1<br>Epidish_CCLE.TIL10_Macrophages.M1<br>CBSX_CCLE.TIL10_Macrophages.M1<br>DeconRNASeq_TIL10_Macrophages.M1
**Macrophages.M1_Subgroup.2.Iteration.1**	DeconRNASeq_LM22_Macrophages.M1<br>CBSX_LM22_Macrophages.M1
**Macrophages.M1_Subgroup.1.Iteration.2**	CBSX_TIL10_Macrophages.M1<br>Macrophages.M1_Subgroup.1.Iteration.1
**Macrophages.M2_Subgroup.1.Iteration.1**	Quantiseq_Macrophages.M2<br>DeconRNASeq_CCLE.TIL10_Macrophages.M2

**Macrophages.M2_Subgroup.2.Iteration.1**	DeconRNASeq_TIL10_Macrophages.M2<br>Epidish_TIL10_Macrophages.M2
**Macrophages.M2_Subgroup.3.Iteration.1**	DeconRNASeq_LM22_Macrophages.M2<br>Epidish_LM22_Macrophages.M2
**Macrophages.M2_Subgroup.1.Iteration.2**	Macrophages.M2_Subgroup.1.Iteration.1<br>Macrophages.M2_Subgroup.2.Iteration.1
**Monocytes_Subgroup.1.Iteration.1**	Epidish_CBSX.NSCLC.PBMCs.scRNAseq_Monocytes<br>CBSX_CBSX.NSCLC.PBMCs.scRNAseq_Monocytes
**Monocytes_Subgroup.2.Iteration.1**	DeconRNASeq_CCLE.TIL10_Monocytes<br>Epidish_TIL10_Monocytes
**Monocytes_Subgroup.4.Iteration.1**	Epidish_LM22_Monocytes<br>CBSX_LM22_Monocytes
**Monocytes_Subgroup.1.Iteration.2**	Epidish_CCLE.TIL10_Monocytes<br>Monocytes_Subgroup.2.Iteration.1
**Monocytes_Subgroup.2.Iteration.2**	CBSX_CCLE.TIL10_Monocytes<br>CBSX_TIL10_Monocytes
**Neutrophils_Subgroup.1.Iteration.1**	MCP_Neutrophils<br>DeconRNASeq_BPRNACan_Neutrophils<br>DeconRNASeq_TIL10_Neutrophils
**Neutrophils_Subgroup.2.Iteration.1**	DeconRNASeq_CCLE.TIL10_Neutrophils<br>Epidish_CCLE.TIL10_Neutrophils<br>CBSX_CCLE.TIL10_Neutrophils
**Neutrophils_Subgroup.3.Iteration.1**	Epidish_LM22_Neutrophils<br>CBSX_LM22_Neutrophils<br>Epidish_TIL10_Neutrophils<br>CBSX_TIL10_Neutrophils
**Neutrophils_Subgroup.1.Iteration.2**	Neutrophils_Subgroup.1.Iteration.1<br>Neutrophils_Subgroup.3.Iteration.1
**NK.cells_Subgroup.1.Iteration.1**	Quantiseq_NK.cells<br>DeconRNASeq_CCLE.TIL10_NK.cells
**NKT.cells_Subgroup.1.Iteration.1**	Epidish_CBSX.NSCLC.PBMCs.scRNAseq_NKT.cells<br>CBSX_CBSX.NSCLC.PBMCs.scRNAseq_NKT.cells
**CD4.cells_Subgroup.1.Iteration.1**	DeconRNASeq_BPRNACan_CD4.cells<br>Epidish_BPRNACanProMet_CD4.cells<br>Epidish_BPRNACan_CD4.cells<br>DeconRNASeq_BPRNACan3DProMet_CD4.cells

**CD4.cells_Subgroup.2.Iteration.1**	DeconRNASeq_CBSX.HNSCC.scRNAseq_CD4.cells<br>Epidish_CBSX.Melanoma.scRNAseq_CD4.cells<br>Epidish_CBSX.HNSCC.scRNAseq_CD4.cells<br>DeconRNASeq_CBSX.Melanoma.scRNAseq_CD4.cells<br>DeconRNASeq_CBSX.NSCLC.PBMCs.scRNAseq_CD4.cells<br>CBSX_CBSX.HNSCC.scRNAseq_CD4.cells<br>CBSX_CBSX.Melanoma.scRNAseq_CD4.cells
**CD4.cells_Subgroup.3.Iteration.1**	DeconRNASeq_CCLE.TIL10_CD4.cells<br>Epidish_CCLE.TIL10_CD4.cells<br>Epidish_TIL10_CD4.cells<br>CBSX_CCLE.TIL10_CD4.cells
**CD4.memory.activated_Subgroup.1.Iteration.1**	DeconRNASeq_LM22_T.cells.CD4.memory.activated<br>CBSX_LM22_T.cells.CD4.memory.activated
**CD8.cells_Subgroup.1.Iteration.1**	MCP_CD8.cells<br>DeconRNASeq_CCLE.TIL10_CD8.cells<br>DeconRNASeq_BPRNACan_CD8.cells<br>Epidish_BPRNACan_CD8.cells<br>DeconRNASeq_CBSX.NSCLC.PBMCs.scRNAseq_CD8.cells
**CD8.cells_Subgroup.2.Iteration.1**	XCell_CD8.cells_central_memory<br>Epidish_CBSX.NSCLC.PBMCs.scRNAseq_CD8.cells
**CD8.cells_Subgroup.3.Iteration.1**	DeconRNASeq_CBSX.HNSCC.scRNAseq_CD8.cells<br>Epidish_LM22_CD8.cells
**CD8.cells_Subgroup.4.Iteration.1**	Epidish_CCLE.TIL10_CD8.cells<br>Epidish_TIL10_CD8.cells<br>CBSX_CCLE.TIL10_CD8.cells
**CD8.cells_Subgroup.1.Iteration.2**	CBSX_LM22_CD8.cells<br>CD8.cells_Subgroup.4.Iteration.1
**CD8.cells_Subgroup.2.Iteration.2**	CD8.cells_Subgroup.1.Iteration.1<br>CD8.cells_Subgroup.2.Iteration.1<br>CD8.cells_Subgroup.3.Iteration.1
**T.cells.regulatory_Subgroup.1.Iteration.1**	DeconRNASeq_CCLE.TIL10_T.cells.regulatory<br>DeconRNASeq_TIL10_T.cells.regulatory<br>Epidish_CCLE.TIL10_T.cells.regulatory
**T.cells.helper_Subgroup.1.Iteration.1**	DeconRNASeq_LM22_T.cells.helper<br>Epidish_LM22_T.cells.helper
**Dendritic.cells_Subgroup.1.Iteration.1**	DeconRNASeq_CBSX.HNSCC.scRNAseq_Dendritic.cells<br>Epidish_CBSX.HNSCC.scRNAseq_Dendritic.cells<br>CBSX_CBSX.HNSCC.scRNAseq_Dendritic.cells
**Dendritic.cells_Subgroup.2.Iteration.1**	Epidish_CCLE.TIL10_Dendritic.cells<br>DeconRNASeq_TIL10_Dendritic.cells

**Cancer_Subgroup.1.Iteration.1**	CBSX_BPRNACan_Cancer<br>CBSX_BPRNACan3DProMet_Cancer<br>DeconRNASeq_BPRNACanProMet_Cancer
**Cancer_Subgroup.2.Iteration.1**	DeconRNASeq_CBSX.HNSCC.scRNAseq_Cancer<br>Epidish_CBSX.HNSCC.scRNAseq_Cancer
**Endothelial_Subgroup.1.Iteration.1**	MCP_Endothelial<br>DeconRNASeq_CBSX.HNSCC.scRNAseq_Endothelial<br>DeconRNASeq_CBSX.Melanoma.scRNAseq_Endothelial<br>CBSX_CBSX.HNSCC.scRNAseq_Endothelial
**Endothelial_Subgroup.1.Iteration.2**	XCell_Endothelial<br>Endothelial_Subgroup.1.Iteration.1
**Endothelial_Subgroup.1.Iteration.3**	Epidish_CBSX.Melanoma.scRNAseq_Endothelial<br>Endothelial_Subgroup.1.Iteration.2
**Plasma.cells_Subgroup.1.Iteration.1**	XCell_B_cell_Plasma.cells<br>DeconRNASeq_LM22_Plasma.cells
**Plasma.cells_Subgroup.1.Iteration.2**	CBSX_LM22_Plasma.cells<br>Plasma.cells_Subgroup.1.Iteration.1
**Mast.resting_Subgroup.1.Iteration.1**	DeconRNASeq_LM22_Mast.resting.cells<br>CBSX_LM22_Mast.resting.cells
**Mast.resting_Subgroup.1.Iteration.2**	Epidish_LM22_Mast.resting.cells<br>Mast.resting_Subgroup.1.Iteration.1
**CAF_Subgroup.1.Iteration.1**	DeconRNASeq_CBSX.HNSCC.scRNAseq_CAF<br>Epidish_CBSX.HNSCC.scRNAseq_CAF<br>CBSX_CBSX.HNSCC.scRNAseq_CAF<br>DeconRNASeq_CBSX.Melanoma.scRNAseq_CAF

Supplementary Table 4: Composition of TF modules obtained from all samples from Lung Predict cohort.

TFs module	Composition
**blue**	AR,ARNT,ASXL1,ATF7,BCLAF1,BHLHE40,BHLHE41,BMAL1,BTG2,CDX2,CEBPA,CLOCK,CREB1,CREB5,DBP,DEAF1,DLX1,E2F7,EBF3,ELF3,ELK4,ESR2,ESRRB,ESRRG,FEV,FOXA2,FOXC2,FOXI1,FOXJ1,FOXL2,FOXO4,FOXP1,FOXP2,GATA4,GATA5,GATA6,GFI1,HDAC4,HESX1,HNF1A,HNF1B,HNF4A,HOXA13,HOXA2,HOXB7,INSM1,IRX1,KAT2B,KDM5B,KLF1,KLF11,KLF15,KLF4,KLF6,KLF7,KLF9,LITAF,LMO4,MAFG,MAFK,MECOM,MEF2B,MEF2C,MLXIP,MNX1,MSX1,MSX2,MXI1,MYF5,MYF6,MYOD1,MYOG,NCOA6,NFIB,NHLH1,NHLH2,NKRF,NPAS2,NR0B1,NR1H3,NR1H4,NR1I3,NR3C1,NR4A1,NR4A2,NR5A1,OLIG1,ONECUT1,PGR,PITX1,PITX3,PLAG1,PLAGL2,POU3F2,PPARA,PPARD,PPARG,PRDM16,PRDM4,PROX1,RB1,RFX2,RFX3,RORC,RXRG,SALL1,SALL4,SIX4,SKIL,SMAD5,SMAD7,SMARCA5,SOX2,SOX4,SPDEF,SRY,TAF1,TBPL1,TBPL2,TBX20,TEAD4,TEF,TFAP2C,TFCP2,TFDP3,TOX,TP73,TRIM28,TSHZ3,TTF1,VHL,VSX1,ZFPM2,ZGLP1,ZIC1,ZNF24

**turquoise**	AHRR,AIP,ARID3B,ARID4B,ATF6,ATOH1,BACH2,BHLHA15,BRD4,CREB3L3,CRX,CTCFL,DLX2,DLX3,DMRT1,DNMT3A,E2F6,ELK1,ELK3,ENO1,ERF,ESRRA,ETV3,FEZF1,FEZF2,FIGLA,FOXA1,FOXD1,FOXD3,FOXM1,FOXN4,FOXO6,GBX2,GCM1,GRHL2,GSC,GTF2IRD1,HDAC1,HDAC3,HES5,HES6,HEY1,HEY2,HIF3A,HIPK2,HIVEP1,HMGB2,HNF4G,HOXA10,HOXA11,HOXB13,HOXB4,HOXB6,HOXC13,HOXD1,HOXD8,HSF2,ID4,IRX4,ISL1,JARID2,KDM5A,KDM5C,KLF12,KLF17,LHX1,LHX2,LHX3,LHX4,LHX8,LMX1A,LMX1B,LYL1,MAFA,MED1,MEIS1,MEIS2,MESP2,MLX,MLXIPL,MTA3,MXD1,MYBL1,NAB2,NANOG,NCOA1,NCOA2,NEUROD1,NEUROD2,NEUROG2,NEUROG3,NFE2,NFE2L1,NFE2L2,NFIA,NFIC,NKX3.2,NKX6.3,NONO,NR1D1,NR1H2,NR1I2,NR2C1,NR2E1,NR5A2,NR6A1,NRL,OTX2,PARK7,PAX4,PAX7,PAX8,PAX9,PDX1,PHOX2A,PHOX2B,PIAS1,POU1F1,POU2F3,POU3F1,POU3F4,POU4F1,POU4F3,POU5F1,PPARGC1A,PPARGC1B,PROP1,PTF1A,PURA,RCOR2,RORB,RREB1,SALL2,SF1,SFPQ,SIM2,SIRT1,SIX3,SMAD6,SMAD9,SMARCC1,SON,SOX3,SP4,SREBF1,SREBF2,SUPT20H,TBP,TBX15,TBX3,TBX6,TCF20,TCF7L1,TEAD2,TFAP2B,TFAP2D,TGIF1,THAP11,TLX2,TRERF1,TRPS1,UBTF,UTF1,VSX2,YBX3,ZBED1,ZBTB14,ZBTB18,ZBTB32,ZBTB33,ZBTB4,ZFHX3,ZMYND8,ZNF202,ZNF217,ZNF219,ZNF236,ZNF331,ZNF335,ZNF350,ZNF382,ZNF423,ZNF436
**yellow**	ARX,ASCL2,ATOH7,BARX1,BARX2,BCL11A,CDX1,CTBP1,CTNNB1,E4F1,EMX2,EN1,ETV1,ETV6,FOXF2,FOXQ1,GATA1,GLI1,GRHL3,GTF2I,HAND1,HAND2,HES1,HHEX,HOXA5,HOXA7,HOXA9,HOXB2,HOXB5,HOXB9,HOXD13,IKZF4,KDM2A,LEF1,LRRFIP1,MAML1,MEF2A,MKX,MYRF,MYT1,MZF1,NFATC4,NKX2.1,NKX6.2,NR1D2,NR2E3,NRG1,NSD2,NUPR1,PAX2,PAX6,PBX1,PBX2,POU4F2,PRDM2,PREB,RARG,RLF,RXRA,SIN3A,SIX1,SOX10,SOX17,SOX18,SOX6,SOX7,SOX9,TBX1,TBX18,TBX5,TBXT,TCF21,TFEB,TGFB1I1,THRA,THRB,TLX3,TSC22D1,YBX1,ZBTB17,ZBTB38,ZBTB7A,ZGPAT,ZNF362,ZNF91
**brown**	ABL1,AIRE,ARID1A,ARID5B,ATF3,BCL11B,BMAL2,BRCA1,CEBPZ,CIITA,CTBP2,CXXC1,EBF1,EGR1,EGR3,ELF2,ERG,ETV2,ETV4,ETV7,FHL2,FOXC1,FOXF1,FOXO3,GATA2,GATA3,GFI1B,GLI3,HBP1,HDGF,HIC1,HIVEP2,HIVEP3,HLX,HOXA1,HOXC6,HR,ID2,IKZF3,IRF6,IRF7,IRF9,JDP2,KAT6A,KAT6B,KLF10,KLF2,MAFB,MAX,MEF2D,MYOCD,NFAT5,NFIL3,NFKBIB,NFYB,NKX2.5,NOTCH1,NOTO,NR2F1,NR2F2,NR4A3,OVOL1,PAX3,PBRM1,PHF5A,PITX2,PKNOX1,PLAGL1,POU2AF1,POU2F1,RFX1,RUNX3,SATB1,SATB2,SMAD2,SMAD3,SMAD4,SNAI1,SP1,SPIC,SRF,TBX10,TCF3,TET1,TFAP4,TFE3,TLX1,TOX3,TP53,TP63,TWIST1,TWIST2,USF1,USF2,VDR,ZBTB16,ZEB2,ZNF354C,ZNF395
**red**	AP1,ATF1,ATF2,BATF,BCL3,BCL6,CEBPB,CEBPD,CEBPG,CREB3,CUX1,DOT1L,EGR2,ELF1,ELF4,EOMES,ETS1,ETS2,ETV5,FLI1,FOS,FOXO1,FOXP3,HMGA1,IKZF1,IKZF2,IRF1,IRF2,IRF3,IRF4,IRF5,IRF8,JUN,JUNB,LMO2,MAF,MITF,MSC,MYB,NFATC1,NFATC2,NFATC3,NFE2L3,NFKB,NFKB1,NFKB2,NFKBIZ,NR2C2,NR3C2,POU2F2,RARA,RBPJ,REL,RELA,RELB,RFX5,RFXANK,RFXAP,RUNX1,SP3,SPI1,SPIB,SSRP1,STAT1,STAT2,STAT3,STAT4,STAT5A,STAT5B,STAT6,TBX21,TCF7,ZXDC
**black**	ARID3A,ARID4A,CHD4,CREB3L1,CREBBP,DACH1,DAXX,E2F1,E2F2,E2F3,E2F4,E2F5,FOXE1,FOXG1,GLI2,HCFC1,HDAC5,HDAC7,HDAC9,HOPX,HOXB3,HSF4,KAT5,KCNIP3,KMT2B,MECP2,MEOX2,MNT,MTF2,MYBL2,MYC,MYCN,NCOR2,NEUROD6,NEUROG1,NFIX,NFYA,NKX6.1,NRF1,OLIG2,PAWR,SALL3,SETBP1,SHOX2,SMARCA1,SMARCA4,SMARCB1,SOX11,SP6,SRSF2,STOX1,TADA2A,TCF15,TFAM,TFDP1,TFDP2,WWTR1,YAP1,YY1,ZFP42,ZFPM1,ZKSCAN7,ZNF143,ZNF160,ZNF76
**green**	AHR,APEX1,ARID1B,ATF4,ATF5,BACH1,BCOR,CEBPE,CREM,CTCF,DLX4,DMAP1,DMTF1,DNMT1,EHF,EPAS1,ESR1,EWSR1,FOSB,FOSL1,FOSL2,FOXA3,FOXH1,GABPA,HIF1A,HLF,HMGA2,HOXD10,HOXD3,HSF1,HTATIP2,ID1,ID3,JUND,KAT7,KDM5D,KLF13,KLF3,KLF5,KLF8,KMT2A,MAZ,MBD2,MEOX1,MIXL1,MTA1,MTF1,NFX1,NR0B2,PA2G4,PATZ1,PAX5,PEG3,PHF20,PIN1,PRRX1,RARB,REST,RXRB,SMAD1,SNAI2,SP2,TAL1,TBR1,TCF7L2,TEAD1,TFAP2A,VENTX,WT1,XBP1,ZBTB7B,ZEB1,ZFX,ZHX2,ZNF148,ZNF263,ZNF300
**pink**	AEBP1,ASCL1,CREBZF,DDIT3,DLX5,DNMT3B,EGR4,ELF5,EP300,EZH2,FGF2,FOXN1,GLIS3,GTF3A,HEYL,HINFP,HOXA4,HOXB1,HOXC8,HOXD4,ING4,IRF2BPL,MBD1,MTA2,NACC1,NCOA3,NCOR1,NFYC,NKX2.2,NKX3.1,NPAS4,NPM1,NR2F6,PAX1,PML,PRDM1,RORA,RUNX2,SCX,SOX5,SP7,TBX2,TCF12,TCF4,VEZF1,ZFP64,ZIC2,ZNF384,ZNF410,ZNF699,ZNF804A

Supplementary Table 5: Composition of deconvolution features subgroups on early stage samples from Lung Predict cohort.

Cell subgroups	Methods-signatures
**B.cells_Subgroup.1.Iteration.1**	Quantiseq_B.cells<br>DeconRNASeq_TIL10_B.cells<br>DeconRNASeq_CCLE.TIL10_B.cells<br>CBSX_TIL10_B.cells

	XCell_B.cells<br>CBSX_CCLE.TIL10_B.cells<br>CBSX_CBSX.Melanoma.scRNAseq_B.cells<br>CBSX_BPRNACan3DProMet_B.cells<br>MCP_B.cells
**B.cells_Subgroup.1.Iteration.2**	DeconRNASeq_BPRNACan3DProMet_B.cells<br>B.cells_Subgroup.1.Iteration.1
**B.cells_Subgroup.2.Iteration.2**	DeconRNASeq_CBSX.HNSCC.scRNAseq_B.cells<br>DeconRNASeq_CBSX.Melanoma.scRNAseq_B.cells
**B.memory_Subgroup.1.Iteration.1**	DeconRNASeq_LM22_B.memory.cells<br>CBSX_LM22_B.memory.cells
**Macrophages.cells_Subgroup.1.Iteration.1**	DeconRNASeq_CBSX.HNSCC.scRNAseq_Macrophages.cells<br>CBSX_CBSX.HNSCC.scRNAseq_Macrophages.cells<br>CBSX_CBSX.Melanoma.scRNAseq_Macrophages.cells
**Macrophages.cells_Subgroup.2.Iteration.1**	DeconRNASeq_CBSX.Melanoma.scRNAseq_Macrophages.cells<br>Epidish_CBSX.Melanoma.scRNAseq_Macrophages.cells
**Macrophages.M0_Subgroup.1.Iteration.1**	Epidish_LM22_Macrophages.M0<br>CBSX_LM22_Macrophages.M0
**Macrophages.M1_Subgroup.1.Iteration.1**	DeconRNASeq_CCLE.TIL10_Macrophages.M1<br>Epidish_CCLE.TIL10_Macrophages.M1<br>DeconRNASeq_TIL10_Macrophages.M1<br>CBSX_CCLE.TIL10_Macrophages.M1
**Macrophages.M1_Subgroup.2.Iteration.1**	DeconRNASeq_LM22_Macrophages.M1<br>CBSX_LM22_Macrophages.M1
**Macrophages.M2_Subgroup.1.Iteration.1**	Quantiseq_Macrophages.M2<br>DeconRNASeq_CCLE.TIL10_Macrophages.M2
**Macrophages.M2_Subgroup.2.Iteration.1**	DeconRNASeq_TIL10_Macrophages.M2<br>Epidish_TIL10_Macrophages.M2
**Macrophages.M2_Subgroup.3.Iteration.1**	DeconRNASeq_LM22_Macrophages.M2<br>Epidish_LM22_Macrophages.M2<br>CBSX_LM22_Macrophages.M2
**Macrophages.M2_Subgroup.1.Iteration.2**	Macrophages.M2_Subgroup.1.Iteration.1<br>Macrophages.M2_Subgroup.2.Iteration.1
**Monocytes_Subgroup.1.Iteration.1**	DeconRNASeq_CBSX.NSCLC.PBMCs.scRNAseq_Monocytes<br>Epidish_CBSX.NSCLC.PBMCs.scRNAseq_Monocytes
**Monocytes_Subgroup.2.Iteration.1**	Epidish_CCLE.TIL10_Monocytes<br>DeconRNASeq_TIL10_Monocytes
**Monocytes_Subgroup.4.Iteration.1**	DeconRNASeq_LM22_Monocytes<br>Epidish_LM22_Monocytes<br>CBSX_LM22_Monocytes
**Monocytes_Subgroup.1.Iteration.2**	CBSX_CBSX.NSCLC.PBMCs.scRNAseq_Monocytes<br>Monocytes_Subgroup.1.Iteration.1
**Monocytes_Subgroup.2.Iteration.2**	CBSX_CCLE.TIL10_Monocytes<br>CBSX_TIL10_Monocytes

**Neutrophils_Subgroup.2.Iteration.1**	DeconRNASeq_CCLE.TIL10_Neutrophils<br>Epidish_CCLE.TIL10_Neutrophils<br>DeconRNASeq_TIL10_Neutrophils<br>CBSX_CCLE.TIL10_Neutrophils
**Neutrophils_Subgroup.3.Iteration.1**	Epidish_TIL10_Neutrophils<br>CBSX_TIL10_Neutrophils
**Neutrophils_Subgroup.1.Iteration.2**	DeconRNASeq_BPRNACan_Neutrophils<br>DeconRNASeq_LM22_Neutrophils
**NK.cells_Subgroup.1.Iteration.1**	DeconRNASeq_CBSX.NSCLC.PBMCs.scRNAseq_NK.cells<br>Epidish_CBSX.NSCLC.PBMCs.scRNAseq_NK.cells
**NK.cells_Subgroup.1.Iteration.2**	DeconRNASeq_CCLE.TIL10_NK.cells<br>DeconRNASeq_TIL10_NK.cells
**CD4.cells_Subgroup.1.Iteration.1**	DeconRNASeq_BPRNACan_CD4.cells<br>Epidish_BPRNACan3DProMet_CD4.cells<br>DeconRNASeq_BPRNACan3DProMet_CD4.cells
**CD4.cells_Subgroup.2.Iteration.1**	DeconRNASeq_CBSX.HNSCC.scRNAseq_CD4.cells<br>DeconRNASeq_CBSX.Melanoma.scRNAseq_CD4.cells<br>Epidish_CBSX.Melanoma.scRNAseq_CD4.cells<br>Epidish_CBSX.HNSCC.scRNAseq_CD4.cells<br>Epidish_CBSX.NSCLC.PBMCs.scRNAseq_CD4.cells
**CD4.cells_Subgroup.3.Iteration.1**	DeconRNASeq_CCLE.TIL10_CD4.cells<br>Epidish_CCLE.TIL10_CD4.cells<br>Epidish_TIL10_CD4.cells
**CD4.memory.activated_Subgroup.1.Iteration.1**	DeconRNASeq_LM22_T.cells.CD4.memory.activated<br>CBSX_LM22_T.cells.CD4.memory.activated
**CD4.memory.resting_Subgroup.1.Iteration.1**	Epidish_LM22_T.cells.CD4.memory.resting<br>CBSX_LM22_T.cells.CD4.memory.resting
**CD8.cells_Subgroup.1.Iteration.1**	MCP_CD8.cells<br>DeconRNASeq_CCLE.TIL10_CD8.cells<br>DeconRNASeq_BPRNACan_CD8.cells
**CD8.cells_Subgroup.2.Iteration.1**	XCell_CD8.cells<br>XCell_CD8.cells_central_memory<br>DeconRNASeq_CBSX.NSCLC.PBMCs.scRNAseq_CD8.cells
**CD8.cells_Subgroup.3.Iteration.1**	CBSX_CBSX.Melanoma.scRNAseq_CD8.cells<br>Epidish_LM22_CD8.cells
**CD8.cells_Subgroup.4.Iteration.1**	Epidish_CCLE.TIL10_CD8.cells<br>CBSX_CCLE.TIL10_CD8.cells<br>CBSX_LM22_CD8.cells
**CD8.cells_Subgroup.1.Iteration.2**	Epidish_BPRNACan_CD8.cells<br>CD8.cells_Subgroup.1.Iteration.1<br>CD8.cells_Subgroup.2.Iteration.1
**CD8.cells_Subgroup.2.Iteration.2**	DeconRNASeq_CBSX.HNSCC.scRNAseq_CD8.cells<br>CD8.cells_Subgroup.3.Iteration.1
**CD8.cells_Subgroup.1.Iteration.3**	CD8.cells_Subgroup.4.Iteration.1<br>CD8.cells_Subgroup.2.Iteration.2
**CD8.cells_Subgroup.1.Iteration.4**	CD8.cells_Subgroup.1.Iteration.2<br>CD8.cells_Subgroup.1.Iteration.3

**T.cells.regulatory_Subgroup.1.Iteration.1**	Quantiseq_T.cells.regulatory<br>Epidish_CCLE.TIL10_T.cells.regulatory
**T.cells.regulatory_Subgroup.3.Iteration.1**	Epidish_TIL10_T.cells.regulatory<br>CBSX_TIL10_T.cells.regulatory
**T.cells.regulatory_Subgroup.1.Iteration.2**	DeconRNASeq_CCLE.TIL10_T.cells.regulatory<br>DeconRNASeq_TIL10_T.cells.regulatory
**T.cells.helper_Subgroup.1.Iteration.1**	DeconRNASeq_LM22_T.cells.helper<br>Epidish_LM22_T.cells.helper
**Cancer_Subgroup.1.Iteration.1**	DeconRNASeq_BPRNACan3DProMet_Cancer<br>Epidish_BPRNACan3DProMet_Cancer<br>CBSX_BPRNACanProMet_Cancer<br>CBSX_BPRNACan3DProMet_Cancer
**Endothelial_Subgroup.1.Iteration.1**	MCP_Endothelial<br>DeconRNASeq_CBSX.Melanoma.scRNAseq_Endothelial<br>CBSX_CBSX.HNSCC.scRNAseq_Endothelial<br>XCell_Endothelial
**Plasma.cells_Subgroup.1.Iteration.1**	XCell_B_cell_Plasma.cells<br>DeconRNASeq_LM22_Plasma.cells
**Plasma.cells_Subgroup.1.Iteration.2**	CBSX_LM22_Plasma.cells<br>Plasma.cells_Subgroup.1.Iteration.1
**Mast.resting_Subgroup.1.Iteration.1**	DeconRNASeq_LM22_Mast.resting.cells<br>CBSX_LM22_Mast.resting.cells<br>Epidish_LM22_Mast.resting.cells
**CAF_Subgroup.1.Iteration.1**	Epidish_CBSX.HNSCC.scRNAseq_CAF<br>Epidish_CBSX.Melanoma.scRNAseq_CAF<br>CBSX_CBSX.Melanoma.scRNAseq_CAF<br>DeconRNASeq_CBSX.Melanoma.scRNAseq_CAF<br>CBSX_CBSX.HNSCC.scRNAseq_CAF

Supplementary Table 6: Composition of TF modules obtained from early stage samples from Lung Predict cohort.

TFs module	Composition
**red**	ARID3A,ARID3B,ARID4A,ASCL1,ATF6,CREB3L1,DACH1,DAXX,DNMT3A,E2F1,E2F2,E2F3,E2F4,E2F5,FIGLA,FOXG1,FOXN4,GLI2,HCFC1,HDAC5,HDAC7,HDAC9,HOPX,HOXA10,HOXB13,HOXB3,HOXC8,HOXD8,HSF4,KMT2B,MBD1,MECP2,MEIS2,MEOX2,MTA2,MTF2,MYBL2,MYC,MYCN,NAB2,NCOR1,NFYA,NKX6.1,NKX6.3,NPAS4,NRF1,PAWR,POU4F1,SALL3,SETBP1,SF1,SHOX2,SIRT1,SMARCA1,SMARCA4,SMARCB1,SOX11,SRSF2,STOX1,TADA2A,TCF7L1,TFAM,TFDP1,UTF1,WWTR1,YAP1,YY1,ZBTB18,ZFPM1,ZKSCAN7,ZNF143,ZNF160,ZNF382,ZNF76
**yellow**	ASCL2,ATOH1,BRD4,CHD4,CREB3L3,CREBBP,CRX,CTCFL,DLX3,DLX5,DMRT1,EGR4,ELK1,ERF,ESRRA,ESRRB,ESRRG,ETV3,FEV,FEZF1,FOXF2,FOXL2,GCM1,GRHL2,GSC,GTF2IRD1,HDAC1,HES6,HIF3A,HIPK2,HIVEP1,HMGB2,HNF4G,HOXB6,HOXC13,HOXD4,ID4,IRX4,ISL1,KDM5A,KDM5C,KLF12,KLF17,LHX1,LHX3,LHX4,LRRFIP1,MAFA,MAML1,MEIS1,MESP2,MXD1,NCOA1,NCOA2,NCOR2,NEUROD1,NEUROD2,NEUROD6,NEUROG1,NEUROG2,NEUROG3,NFIC,NFIX,NR1D2,NR1I2,NR2C1,NR4A1,NR5A2,NR6A1,NRL,PAX4,PAX7,PDX1,PHOX2A,PHOX2B,PML,POU1F1,POU2F3,POU3F4,POU4F2,POU5F1,PPARGC1A,PPARGC1B,PROP1,PTF1A,RORA,RREB1,SALL2,SIM2,SMARCC1,SOX3,SP4,TBX15,TBX3,TCF15,TGIF1,THAP11,THRA,TLX2,TRERF1,TRPS1,ZBTB14,ZBTB32,ZFP42,ZMYND8,ZNF202,ZNF217,ZNF236,ZNF350,ZNF436,ZNF699

**blue**	ARID1A,ARID5B,ARNT,ARX,ASXL1,BARX1,BCL11A,BCL11B,BHLHE40,BHLHE41,BMAL1,BTG2,CDX1,CEBPA,CLOCK,CREM,CTBP1,CTNNB1,DEAF1,DLX1,DNMT1,EBF3,ELF3,ERG,ESR2,ETV6,FOXC2,FOXF1,FOXI1,FOXO3,FOXO4,FOXP1,FOXP2,FOXQ1,GATA1,GATA2,GATA4,GATA6,GFI1,GLI1,GRHL3,GTF2I,HBP1,HDAC4,HESX1,HHEX,HLF,HMGA2,HOXA11,HOXA13,HOXA2,HOXA5,HOXA9,HOXB7,HOXB9,HOXC6,HOXD13,IKZF3,IKZF4,KDM5B,KLF1,KLF11,KLF15,KLF2,KLF6,KLF7,KLF9,MAFG,MAX,MEF2B,MEF2D,MKX,MLXIP,MLXIPL,MNX1,MSX2,MYF5,MYOCD,MYT1,MZF1,NFAT5,NFATC4,NKX2.5,NKX3.2,NR0B1,NR1H3,NR1H4,NR5A1,NRG1,OLIG1,OVOL1,PAX2,PAX3,PBRM1,PBX1,PBX2,PGR,PITX3,PLAGL1,POU3F2,PPARD,PRDM16,PRDM2,RARB,RARG,RB1,RFX1,RFX2,RXRB,SIX1,SMAD5,SMAD6,SMAD7,SOX7,SOX9,SRY,TAF1,TBX1,TBX20,TBX6,TCF21,TEF,TFAP2C,TFCP2,TFDP3,TFEB,TGFB1I1,TOX,TOX3,TP53,TP63,TP73,TRIM28,TTF1,VHL,ZBTB16,ZFPM2,ZGLP1,ZHX2,ZNF24,ZNF335,ZNF362,ZNF423,ZNF91
**green**	AR,ARID4B,ATF7,BACH2,CDX2,CREB5,DBP,DLX2,E2F6,E2F7,ELK3,ELK4,ENO1,FOXA1,FOXA2,FOXD3,FOXJ1,GATA5,HEY1,HEY2,HNF1A,HNF1B,HNF4A,HOXB4,HSF2,ING4,INSM1,IRX1,JARID2,KAT2B,KLF4,LHX8,LMO4,LYL1,MECOM,MED1,MEF2C,MSX1,MTA3,MXI1,MYF6,MYOD1,MYOG,NCOA6,NFE2L2,NFIA,NHLH1,NHLH2,NKRF,NONO,NPAS2,NR1H2,NR1I3,NR3C1,NR4A2,ONECUT1,OTX2,PITX1,PLAG1,PLAGL2,POU3F1,POU4F3,PPARA,PPARG,PRDM4,PROX1,PURA,RCOR2,RFX3,RXRG,SALL1,SALL4,SFPQ,SIX4,SKIL,SMAD9,SMARCA5,SOX2,SOX4,SPDEF,SREBF1,SREBF2,SUPT20H,TBPL2,TCF20,TEAD2,TSHZ3,VSX1,VSX2,YBX3,ZBTB33,ZFHX3,ZNF331
**turquoise**	ABL1,AHRR,AIP,APEX1,ATF5,ATOH7,BARX2,BCLAF1,BHLHA15,BRCA1,CTCF,DDIT3,DMAP1,DMTF1,DNMT3B,E4F1,ELF5,EMX2,EN1,EP300,EZH2,FEZF2,FGF2,FOXA3,FOXD1,FOXE1,FOXH1,FOXM1,FOXN1,FOXO6,GBX2,GLIS3,GTF3A,HAND1,HAND2,HDAC3,HES1,HES5,HEYL,HINFP,HOXA4,HOXA7,HOXB1,HOXB2,HOXB5,HOXD1,HOXD10,HSF1,ID3,IRF2BPL,KAT5,KCNIP3,KDM2A,KLF13,KLF8,LEF1,LHX2,LITAF,LMX1A,LMX1B,MAFK,MAZ,MBD2,MEF2A,MEOX1,MIXL1,MLX,MNT,MTF1,MYBL1,MYRF,NACC1,NANOG,NCOA3,NFE2,NFE2L1,NFIB,NFYC,NKX2.1,NKX2.2,NKX3.1,NKX6.2,NPM1,NR0B2,NR1D1,NR2E1,NR2E3,NR2F6,NSD2,NUPR1,OLIG2,PA2G4,PARK7,PATZ1,PAX1,PAX6,PAX8,PAX9,PEG3,PIAS1,PREB,PRRX1,RLF,RORB,RORC,RUNX2,RXRA,SCX,SIN3A,SIX3,SNAI2,SON,SOX10,SOX17,SOX18,SOX5,SOX6,SP2,SP6,TBP,TBPL1,TBR1,TBX2,TBX5,TBXT,TCF12,TCF4,TEAD1,TEAD4,TFAP2D,TFDP2,THRB,TSC22D1,UBTF,VENTX,VEZF1,WT1,YBX1,ZBED1,ZBTB17,ZBTB38,ZBTB4,ZBTB7A,ZBTB7B,ZFP64,ZFX,ZGPAT,ZIC1,ZIC2,ZNF148,ZNF219,ZNF384,ZNF410,ZNF804A
**black**	AIRE,ATF1,ATF2,ATF3,BCL3,BCL6,BMAL2,CEBPB,CEBPD,CEBPG,DLX4,EGR1,EGR3,ELF2,ETV2,ETV4,FLI1,FOSL2,FOXC1,FOXO1,FOXP3,GATA3,GFI1B,GLI3,HIC1,HIF1A,IKZF1,JDP2,JUND,KAT6A,KLF10,KLF5,LMO2,MAFB,MITF,MSC,NFATC3,NFE2L3,NFIL3,NOTCH1,NR2F1,NR2F2,NR3C2,PITX2,PKNOX1,RARA,RUNX1,SMAD1,SMAD2,SMAD3,SMAD4,SNAI1,SP1,SRF,SSRP1,STAT5B,STAT6,TAL1,TFAP2A,TLX3,TWIST1,USF1,USF2,ZNF354C
**brown**	AEBP1,AHR,AP1,ARID1B,ATF4,BACH1,BATF,BCOR,CEBPE,CEBPZ,CIITA,CREB1,CREB3,CREBZF,CTBP2,CUX1,CXXC1,DOT1L,EBF1,EGR2,EHF,ELF1,ELF4,EOMES,EPAS1,ESR1,ETS1,ETS2,ETV1,ETV5,ETV7,EWSR1,FHL2,FOS,FOSB,FOSL1,GABPA,HDGF,HIVEP2,HIVEP3,HLX,HMGA1,HOXA1,HOXD3,HR,HTATIP2,ID1,ID2,IKZF2,IRF1,IRF2,IRF3,IRF4,IRF5,IRF6,IRF7,IRF8,IRF9,JUN,JUNB,KAT6B,KAT7,KDM5D,KLF3,KMT2A,MAF,MTA1,MYB,NFATC1,NFATC2,NFKB,NFKB1,NFKB2,NFKBIB,NFKBIZ,NFX1,NFYB,NOTO,NR2C2,NR4A3,PAX5,PHF20,PHF5A,PIN1,POU2AF1,POU2F1,POU2F2,PRDM1,RBPJ,REL,RELA,RELB,REST,RFX5,RFXANK,RFXAP,RUNX3,SATB1,SATB2,SP3,SP7,SPI1,SPIB,SPIC,STAT1,STAT2,STAT3,STAT4,STAT5A,TBX10,TBX18,TBX21,TCF3,TCF7,TCF7L2,TET1,TFAP2B,TFAP4,TFE3,TLX1,TWIST2,VDR,XBP1,ZEB1,ZEB2,ZNF263,ZNF300,ZNF395,ZXDC

Supplementary Table 7: Composition of cell groups obtained from early stage samples from Lung Predict cohort.

Cell groups	Methods-signatures
**Dendrogram_red_turquoise_black_brown.group_1**	Epidish_CBSX.NSCLC.PBMCs.scRNAseq_B.cells<br>Cancer_Subgroup.1.Iteration.1<br>Epidish_LM22_B.naive.cells<br>CBSX_CBSX.NSCLC.PBMCs.scRNAseq_B.cells<br>CBSX_LM22_B.naive.cells<br>CBSX_TIL10_Dendritic.cells<br>Epidish_CBSX.HNSCC.scRNAseq_Dendritic.cells<br>CBSX_LM22_Dendritic.activated.cells

**Dendrogram_red_turquoise_black_brown.group_2**	DeconRNASeq_CBSX.Melanoma.scRNAseq_Cancer<br>DeconRNASeq_CBSX.HNSCC.scRNAseq_Myocytes<br>MCP_Neutrophils<br>CBSX_TIL10_Macrophages.M2<br>Monocytes_Subgroup.1.Iteration.2
**Dendrogram_red_turquoise_black_brown.group_3**	Epidish_LM22_NK.resting<br>CBSX_LM22_NK.resting<br>Epidish_TIL10_Macrophages.M1<br>DeconRNASeq_LM22_Macrophages.M0<br>Macrophages.M1_Subgroup.2.Iteration.1
**Dendrogram_red_turquoise_black_brown.group_4**	CBSX_CCLE.TIL10_Cancer<br>DeconRNASeq_CBSX.NSCLC.PBMCs.scRNAseq_NKT.cells<br>MCP_Fibroblasts<br>CAF_Subgroup.1.Iteration.1<br>B.cells_Subgroup.2.Iteration.2<br>Plasma.cells_Subgroup.1.Iteration.2<br>DeconRNASeq_CBSX.Melanoma.scRNAseq_NK.cells<br>CBSX_CBSX.HNSCC.scRNAseq_Dendritic.cells<br>XCell_T_cell_CD4.cells._Th2<br>XCell_T_cell_CD4.cells._Th1<br>DeconRNASeq_CBSX.HNSCC.scRNAseq_Cancer
**Dendrogram_red_turquoise_black_brown.group_7**	Endothelial_Subgroup.1.Iteration.1<br>DeconRNASeq_BPRNACanProMet_Macrophages.M2<br>NK.cells_Subgroup.1.Iteration.2<br>CBSX_CCLE.TIL10_Macrophages.M2<br>Macrophages.M2_Subgroup.1.Iteration.2<br>DeconRNASeq_LM22_Dendritic.activated.cells<br>Macrophages.cells_Subgroup.2.Iteration.1<br>Macrophages.M2_Subgroup.3.Iteration.1<br>Quantiseq_NK.cells<br>DeconRNASeq_LM22_Dendritic.resting.cells<br>CD4.cells_Subgroup.1.Iteration.1<br>CD4.memory.resting_Subgroup.1.Iteration.1<br>DeconRNASeq_CBSX.HNSCC.scRNAseq_Mast.cells
**Dendrogram_red_turquoise_black_brown.group_8**	Monocytes_Subgroup.2.Iteration.2<br>CBSX_CCLE.TIL10_CD4.cells<br>Mast.resting_Subgroup.1.Iteration.1<br>Epidish_CBSX.Melanoma.scRNAseq_Endothelial<br>Monocytes_Subgroup.2.Iteration.1<br>Neutrophils_Subgroup.2.Iteration.1<br>Monocytes_Subgroup.4.Iteration.1<br>MCP_Dendritic.cells
**Dendrogram_red_turquoise_black_brown.group_9**	DeconRNASeq_BPRNACan3DProMet_Monocytes<br>Quantiseq_Neutrophils<br>CD4.cells_Subgroup.2.Iteration.1<br>Macrophages.cells_Subgroup.1.Iteration.1<br>DeconRNASeq_LM22_T.cells.CD4.memory.resting<br>CBSX_CBSX.Melanoma.scRNAseq_CD4.cells<br>CD4.cells_Subgroup.3.Iteration.1<br>Epidish_CCLE.TIL10_NK.cells<br>XCell_CAF
**Dendrogram_yellow_blue_green.group_1**	DeconRNASeq_CBSX.HNSCC.scRNAseq_Mast.cells<br>CD4.cells_Subgroup.1.Iteration.1<br>CD4.memory.resting_Subgroup.1.Iteration.1<br>Endothelial_Subgroup.1.Iteration.1<br>Monocytes_Subgroup.1.Iteration.2<br>Neutrophils_Subgroup.3.Iteration.1<br>DeconRNASeq_LM22_Dendritic.activated.cells

**Dendrogram_yellow_blue_green.group_2**	Epidish_CCLE.TIL10_NK.cells<br>Quantiseq_Neutrophils<br>CD4.cells_Subgroup.2.Iteration.1<br>DeconRNASeq_LM22_T.cells.CD4.memory.resting<br>CBSX_CCLE.TIL10_CD4.cells<br>XCell_CAF<br>DeconRNASeq_LM22_Dendritic.resting.cells<br>CBSX_BPRNACanProMet_CD4.cells<br>CD4.cells_Subgroup.3.Iteration.1<br>DeconRNASeq_BPRNACanProMet_Macrophages.M2<br>Mast.resting_Subgroup.1.Iteration.1<br>Epidish_CBSX.Melanoma.scRNAseq_Endothelial<br>Monocytes_Subgroup.2.Iteration.2<br>CBSX_CBSX.Melanoma.scRNAseq_CD4.cells<br>DeconRNASeq_BPRNACan3DProMet_Monocytes<br>Neutrophils_Subgroup.2.Iteration.1<br>MCP_Dendritic.cells<br>Monocytes_Subgroup.2.Iteration.1<br>Monocytes_Subgroup.4.Iteration.1
**Dendrogram_yellow_blue_green.group_3**	B.memory_Subgroup.1.Iteration.1<br>CBSX_TIL10_NK.cells<br>Quantiseq_Macrophages.M1<br>XCell_Myeloid_Dendritic.activated.cells<br>Macrophages.M2_Subgroup.3.Iteration.1<br>Epidish_CBSX.NSCLC.PBMCs.scRNAseq_CD8.cells<br>Macrophages.M1_Subgroup.1.Iteration.1<br>CBSX_CBSX.HNSCC.scRNAseq_CD4.cells<br>T.cells.regulatory_Subgroup.1.Iteration.1
**Dendrogram_yellow_blue_green.group_4**	Macrophages.M1_Subgroup.2.Iteration.1<br>B.cells_Subgroup.1.Iteration.2<br>CD8.cells_Subgroup.1.Iteration.4<br>Epidish_TIL10_NK.cells<br>CD4.memory.activated_Subgroup.1.Iteration.1<br>XCell_T_cell_CD4.cells._Th2<br>Epidish_CBSX.HNSCC.scRNAseq_Dendritic.cells<br>DeconRNASeq_CBSX.Melanoma.scRNAseq_NK.cells<br>CBSX_TIL10_CD8.cells<br>DeconRNASeq_BPRNACanProMet_Macrophages.M1<br>CBSX_CBSX.NSCLC.PBMCs.scRNAseq_B.cells<br>DeconRNASeq_LM22_NK.resting
**Dendrogram_yellow_blue_green.group_5**	B.cells_Subgroup.2.Iteration.2<br>Plasma.cells_Subgroup.1.Iteration.2<br>CBSX_CCLE.TIL10_Cancer<br>DeconRNASeq_LM22_Macrophages.M0<br>DeconRNASeq_CBSX.HNSCC.scRNAseq_Cancer<br>CBSX_LM22_NK.resting<br>CAF_Subgroup.1.Iteration.1
**Dendrogram_yellow_blue_green.group_6**	XCell_T_cell_NK.cells<br>XCell_T_cell_CD4.cells._Th1<br>Cancer_Subgroup.1.Iteration.1<br>Epidish_CBSX.NSCLC.PBMCs.scRNAseq_B.cells<br>DeconRNASeq_CBSX.NSCLC.PBMCs.scRNAseq_NKT.cells<br>MCP_Neutrophils<br>DeconRNASeq_CBSX.Melanoma.scRNAseq_Cancer<br>DeconRNASeq_CBSX.HNSCC.scRNAseq_Myocytes<br>Epidish_LM22_B.naive.cells<br>Neutrophils_Subgroup.1.Iteration.2

**Dendrogram_red_turquoise_black_brown.group_combined_1**	B.cells_Subgroup.1.Iteration.2<br>Quantiseq_Macrophages.M1<br>CBSX_TIL10_NK.cells<br>CD4.memory.activated_Subgroup.1.Iteration.1<br>CD8.cells_Subgroup.1.Iteration.4<br>DeconRNASeq_BPRNACanProMet_Macrophages.M1<br>CBSX_TIL10_Macrophages.M1<br>Macrophages.M1_Subgroup.1.Iteration.1<br>T.cells.regulatory_Subgroup.1.Iteration.1<br>DeconRNASeq_CBSX.HNSCC.scRNAseq_CAF<br>Epidish_CBSX.NSCLC.PBMCs.scRNAseq_CD8.cells<br>CBSX_CBSX.HNSCC.scRNAseq_CD4.cells<br>XCell_Myeloid_Dendritic.activated.cells<br>CBSX_CCLE.TIL10_NK.cells<br>CBSX_BPRNACanProMet_CD4.cells<br>CBSX_CBSX.NSCLC.PBMCs.scRNAseq_CD4.cells<br>Epidish_CCLE.TIL10_B.cells<br>Macrophages.M0_Subgroup.1.Iteration.1

Supplementary Table 8: Differential expression markers between NK peripheral (pct.1) and NK dysfunctional (pct.2) (p_val_adj <0.05 and abs(avg_log2FC) > 1)

	p_val	avg_log2FC	pct.1	pct.2	p_val_adj
**FGFBP2**	2.08460609594769E-101	1.89548593371525	0.77	0.187	4.98325087236295E-97
**FCGR3A**	2.68967604357574E-92	1.85860456526627	0.725	0.175	6.42967058216781E-88
**GZMK**	2.44395722196532E-74	-2.33065401366759	0.112	0.538	5.8422797391081E-70
**GNLY**	7.50501944505041E-68	1.09828833777124	0.959	0.592	1.7940748983393E-63
**ADGRG1**	2.47427509078513E-57	1.22630997552031	0.585	0.156	5.91475460452186E-53
**GZMB**	4.88309916799668E-53	1.13478919437545	0.855	0.459	1.16730485610961E-48
**SPON2**	3.14262118111522E-49	1.58190278387076	0.567	0.193	7.51243593345592E-45
**PRF1**	1.02985632956447E-42	1.10490490284788	0.816	0.541	2.46187155582386E-38
**DUSP4**	1.42375033095113E-40	-1.54951702436544	0.038	0.284	3.40347516613867E-36
**MYOM2**	3.24330456353166E-32	1.50511093513896	0.242	0.017	7.75311955912243E-28
**IL7R**	3.97913707161018E-32	-1.30346073474289	0.204	0.491	9.51212716968414E-28
**RGS1**	1.44246574665375E-20	-1.03999986890825	0.246	0.459	3.4482143673758E-16
**RGCC**	1.82451899463353E-18	-1.2214217217114	0.222	0.417	4.36151265667146E-14
**S100B**	8.00224023992364E-14	1.23423795899411	0.168	0.042	1.91293552935375E-09
**LMNA**	9.39433297169988E-12	-1.09484727832967	0.119	0.25	2.24571529688486E-07

Supplementary Table 9: Differential expression markers between NK peripheral (pct.1) and NK Tissue resident (pct.2) (p_val_adj <0.05 and abs(avg_log2FC) > 1)

	p_val	avg_log2FC	pct.1	pct.2	p_val_adj
**FGFBP2**	1.00730450013112E-150	2.93590399923422	0.77	0.023	2.40796140756344E-146
**DUSP4**	2.92275475076596E-149	-2.88137756370488	0.038	0.691	6.98684523170603E-145
**FCGR3A**	1.5205232452284E-120	2.52130152975208	0.725	0.078	3.6348108177185E-116
**NKG7**	7.29914390298183E-105	1.11532402772826	0.998	0.94	1.74486035000781E-100
**LDLRAD4**	4.84474666637011E-86	-2.08322398425996	0.036	0.47	1.15813669059578E-81
**SPON2**	5.84794130622426E-86	2.39843237324021	0.567	0.058	1.39795036925291E-81
**GAPDH**	1.4234825547175E-85	-1.40150633484763	0.903	0.982	3.40283504705218E-81
**KIR2DL4**	2.54087858778996E-81	-1.68967104991992	0.066	0.514	6.0739702641119E-77
**KLF2**	1.58444701943375E-80	1.52263761803298	0.783	0.299	3.78762059995638E-76
**RGCC**	2.17901938859824E-80	-2.07151060190251	0.222	0.705	5.2089458484441E-76
**LGALS3**	7.39295778233682E-80	-1.49983829307337	0.077	0.53	1.76728655786762E-75
**KRT86**	4.53492869940734E-79	-2.15796373532198	0.014	0.399	1.08407470559332E-74
**COTL1**	7.18335478998067E-72	-1.52044567661642	0.255	0.703	1.71718096254488E-67
**TXNIP**	2.80590321755904E-71	1.56579714639654	0.847	0.528	6.70751164157488E-67
**CAPG**	4.89473582493126E-71	-1.27950319342389	0.019	0.378	1.17008659894982E-66
**GZMH**	5.37968846263582E-69	1.34307749505042	0.867	0.565	1.28601452699309E-64
**ZNF683**	1.68526022129167E-66	-1.71136665086086	0.075	0.468	4.02861455899773E-62
**ITM2C**	3.62472722036091E-65	-1.34091628314668	0.021	0.364	8.66491042027275E-61
**PLAC8**	4.12717867451217E-64	1.59056030995204	0.509	0.078	9.86602062142135E-60
**VIM**	2.86516457015107E-63	-1.63198702219667	0.751	0.949	6.84917590494613E-59
**S1PR5**	3.70346693635634E-63	1.58094930650611	0.429	0.021	8.85313771135983E-59
**ADGRG1**	1.01744080366623E-61	1.45762645351272	0.585	0.15	2.43219224116413E-57
**RBPJ**	2.25171527574783E-61	-1.24895902362193	0.086	0.472	5.38272536667519E-57

**CSF1**	3.59081537078718E-61	-1.86459574369848	0.018	0.336	8.58384414386676E-57
**CD7**	4.69125012168749E-59	-1.15541362336368	0.681	0.894	1.12144334158939E-54
**GEM**	5.96432091975882E-59	-1.470477240972	0.01	0.306	1.42577091586834E-54
**ITGB2**	2.62961887096676E-57	1.11047863365423	0.846	0.601	6.28610391104603E-53
**SAMSN1**	1.89151414851524E-54	-1.23234565144592	0.263	0.671	4.52166457202569E-50
**LINC02446**	1.04303830871202E-53	-2.03949023866877	0.16	0.518	2.49338307697609E-49
**KLRG1**	1.60188595358552E-52	1.38134702289025	0.56	0.17	3.82930837204618E-48
**SIRPG**	1.88161555207666E-51	-1.05534812362569	0.025	0.311	4.49800197723925E-47
**EFHD2**	1.22181078337035E-50	1.34491662705177	0.551	0.178	2.92073867764682E-46
**PLP2**	1.83674829412564E-50	-1.05544493834848	0.147	0.527	4.39074679710735E-46
**ALOX5AP**	2.55218516192026E-50	-1.22073100263921	0.311	0.691	6.10099862957037E-46
**PLEK**	9.01462209064204E-50	1.35091691287384	0.498	0.125	2.15494541076798E-45
**HLA-DRA**	9.5164814978793E-50	-1.31874531261554	0.095	0.435	2.27491490206805E-45
**CXCR6**	1.33955340106251E-48	-1.21072584162458	0.013	0.269	3.20220240523994E-44
**RGS1**	2.53633746234135E-48	-1.17015443877887	0.246	0.64	6.06311470372699E-44
**CTSA**	2.85745931287537E-48	-1.03625251435498	0.104	0.449	6.83075648742857E-44
**GZMM**	3.5340065510414E-46	1.09242795155489	0.701	0.38	8.44804266026447E-42
**SPRY1**	5.10161120930779E-46	-1.10087978303947	0.013	0.258	1.21954015958503E-41
**CD44**	5.23320617244331E-45	-1.02932658976963	0.462	0.774	1.25099793552257E-40
**KLRC1**	1.44683102335442E-44	-1.45560131256627	0.063	0.359	3.45864956132873E-40
**KLRC2**	5.35899066100842E-43	-1.12435109099194	0.249	0.624	1.28106671751406E-38
**TNFRSF18**	8.33054360169164E-43	-1.19682855643372	0.064	0.353	1.99141644798439E-38
**KLRF1**	2.42217807457388E-42	1.33468972926496	0.434	0.097	5.79021668726886E-38
**CRIP1**	6.79066805079546E-42	-1.30158656639898	0.737	0.906	1.62330919754265E-37
**GALNT2**	2.18025483380709E-41	-1.02700196861867	0.054	0.327	5.21189918021586E-37
**CCND3**	2.45453378148759E-41	1.05774050213402	0.662	0.367	5.86756300464608E-37
**ITGAE**	3.32421392129839E-41	-1.01525554141991	0.119	0.436	7.94653337886381E-37

**PRSS23**	4.99593915342317E-41	1.2090114673941	0.293	0.012	1.19427925462581E-36
**PHLDA1**	1.11379189539802E-40	-1.19894872259238	0.138	0.456	2.66251952594896E-36
**KRT81**	1.61182739036426E-39	-1.13471020159354	0.006	0.208	3.85307337666575E-35
**GZMK**	6.20823379888751E-39	-1.35722810614402	0.112	0.415	1.48407828962406E-34
**TNF**	9.97230531975504E-39	1.80589580508167	0.515	0.193	2.38387958668744E-34
**XCL1**	1.79272444090836E-38	-2.16031930647756	0.226	0.532	4.28550777599144E-34
**BIN2**	2.26764846129845E-38	1.11101901111525	0.601	0.314	5.42081364673394E-34
**MYOM2**	2.21221272926112E-36	1.62742792275453	0.242	0	5.28829452929871E-32
**LMNA**	1.01821956512296E-34	-1.77990917847407	0.119	0.398	2.43405387042643E-30
**TRDV1**	7.09119900201194E-34	-1.67266258281495	0.045	0.269	1.69515112143095E-29
**HLA-DRB1**	1.10995672550228E-32	-1.19627719190046	0.368	0.638	2.65335155231321E-28
**SYNE1**	1.7171234783739E-30	1.08417836242332	0.422	0.154	4.10478367505282E-26
**MYBL1**	2.80961622885085E-30	1.00378392438919	0.274	0.039	6.71638759506796E-26
**TTC38**	8.41527068876505E-30	1.0070229908877	0.354	0.097	2.01167045814928E-25
**FABP5**	3.52369930304876E-29	-1.04717729460883	0.092	0.33	8.42340318393805E-25
**ID3**	1.00727601971069E-28	-1.21833514474547	0.049	0.253	2.4078933251184E-24
**ISG15**	4.22269828050077E-27	-1.01234086837906	0.18	0.443	1.00943602395371E-22
**S100B**	2.0871207179655E-22	1.56820910501893	0.168	0.007	4.98926207629652E-18
**ATF3**	3.77140668193021E-20	-1.0205028325791	0.13	0.339	9.01554767315417E-16
**HSPB1**	2.07046829618126E-14	-1.44486318729009	0.379	0.565	4.94945446202129E-10
**BAG3**	2.30802996488002E-14	-1.61509526126813	0.088	0.226	5.51734563104569E-10
**TRBV9**	5.80826145966367E-14	1.24718898530557	0.106	0.005	1.3884649019326E-09
**SERPINH1**	3.62779139995261E-11	-1.17190773173246	0.033	0.124	8.67223534158671E-07

Supplementary Table 10: Differential expression markers between NK Dysfunctional (pct.1) and NK Tissue resident (pct.2) (p_val_adj <0.05 and abs(avg_log2FC) > 1)

	p_val	avg_log2FC	pct.1	pct.2	p_val_adj
**CD7**	6.32687370520863E-58	-1.23376817954073	0.59	0.894	1.51243915923012E-53
**GAPDH**	1.0081625922189E-55	-1.17440736124193	0.882	0.982	2.41001267669928E-51
**KIR2DL4**	4.35405923335768E-55	-1.44715130466385	0.084	0.514	1.04083785973415E-50
**KRT86**	1.29735129194966E-54	-2.040926439276	0.027	0.399	3.10131826340566E-50
**KLRC2**	5.01767718473208E-51	-1.27275107685976	0.161	0.624	1.1994757310102E-46
**DUSP4**	1.89081856743242E-43	-1.33186053933944	0.284	0.691	4.5200017854472E-39
**LGALS3**	4.39981046341498E-40	-1.09727350446499	0.153	0.53	1.05177469127935E-35
**LDLRAD4**	4.67886840878544E-38	-1.2813017340718	0.124	0.47	1.11848349312016E-33
**ZNF683**	2.07895218924443E-37	-1.27937133564168	0.121	0.468	4.9697352083888E-33
**RBPJ**	1.62761810646781E-35	-1.03734789994945	0.131	0.472	3.89082108351129E-31
**LINC02446**	6.00613491357105E-35	-1.54315678326404	0.183	0.518	1.43576655108916E-30
**GZMB**	1.17798721284482E-33	-1.30516897228585	0.459	0.776	2.81597843230555E-29
**GEM**	5.81142744616175E-33	-1.23955326242761	0.039	0.306	1.38922173100497E-28
**CSF1**	1.21640425950476E-32	-1.45328769023356	0.055	0.336	2.90781438234614E-28
**TRDC**	6.6403090008078E-31	-1.0209743719258	0.143	0.472	1.5873658666431E-26
**KLRC1**	9.57256822819788E-27	-1.12551659535456	0.092	0.359	2.2883224349507E-22
**KLF2**	5.85828027369389E-25	1.13508510591728	0.548	0.299	1.40042189942652E-20
**KRT81**	1.15113776399075E-24	-1.03627813008372	0.018	0.208	2.75179482481988E-20
**TRDV1**	1.33227599371944E-23	-1.56079345498943	0.055	0.269	3.18480576298631E-19
**KLRG1**	3.61571330554947E-23	1.07844084726384	0.413	0.17	8.64336265691602E-19
**CRIP1**	6.95861125654981E-23	-1.00710661099701	0.724	0.906	1.66345602087823E-18
**FGFBP2**	1.00321265054699E-19	1.04041806551897	0.187	0.023	2.39817984113258E-15
**KIR2DL3**	1.65570214464406E-19	-1.06276033082207	0.052	0.239	3.95795597677161E-15
**TXNIP**	1.82096701178912E-19	1.12794575088879	0.674	0.528	4.35302164168189E-15
**KIR3DL1**	8.18384355057614E-18	-1.07396285748355	0.039	0.201	1.95634780076523E-13
**TNF**	1.42798349025974E-17	1.29739855915977	0.407	0.193	3.4135945334659E-13

**XCL2**	2.20392401597922E-15	-1.09945617749461	0.422	0.618	5.26848036019832E-11
**XCL1**	1.22692629916812E-10	-1.1821445501095	0.366	0.532	2.9329673181614E-06
**BAG3**	4.35357242696382E-07	-1.26805884288246	0.119	0.226	0.010407214886657

References

Aran D, Hu Z, Butte A. xCell: digitally portraying the tissue cellular heterogeneity landscape. Genome Biol. (2017) 18:220. doi: 10.1186/s13059-017-1349-1

Ayers M, Lunceford J, Nebozhyn M, et al. IFN-g–related mRNA profile predicts clinical response to PD-1 blockade. J Clin Invest. (2017) 127:2930–40. doi: 10.1172/JCI91190

Becht E, Giraldo NA, Lacroix L, et al. Estimating the population abundance of tissue-infiltrating immune and stromal cell populations using gene expression. Genome Biol. (2016) 17:218. doi: 10.1186/s13059-016-1070-5

Cabrita R, Lauss M, Sanna A, et al. Tertiary lymphoid structures improve immunotherapy and survival in melanoma. Nature. (2020) 577:561–5. doi: 10.1038/S41586-019-1914-8

Davoli T, Uno H, Wooten EC, Elledge SJ. Tumor aneuploidy correlates with markers of immune evasion and with reduced response to immunotherapy. Science.(2017) 355:eaaf8399. doi: 10.1126/science.aaf8399

Finotello F, Mayer C, Plattner C, Laschober G, et al. Molecular and pharmacological modulators of the tumor immune contexture revealed by deconvolution of RNA-seq data. Genome Med. (2019) 11:34. doi: 10.1186/s13073-019-0638-6

Gong T, Szustakowski JD. DeconRNASeq: a statistical framework for deconvolution of heterogeneous tissue samples based on mRNA-Seq data. Bioinformatics. (2013) 29:1083–5. doi: 10.1093/bioinformatics/btt090

Jerby-Arnon L, Shah P, Cuoco MS, et al. A cancer cell program promotes T cell exclusion and resistance to checkpoint blockade. Cell. (2018) 175:984–97. doi: 10.1016/J.cell.2018.09.006

Messina JL, Fenstermacher DA, Eschrich S, Qu X, Berglund AE, Lloyd MC, et al. 12-Chemokine gene signature identifies lymph node-like structures in melanoma: potential for patient selection for immunotherapy? Sci Rep. (2012) 2:765. doi: 10.1038/Srep00765

Newman A, Steen C, Liu C, et al. Determining cell type abundance and expression from bulk tissues with digital cytometry. Nat Biotech. (2019) 37:773–82. doi: 10.1038/s41587-019-0114-2

Roh W, Chen P, Reuben A, et al. Integrated molecular analysis of tumor biopsies on sequential CTLA-4 and PD-1 blockade reveals markers of response and resistance. Sci Transl Med. (2017) 9. doi: 10.1126/scitranslmed.aah3560

Rooney MS, Shukla SA, Wu CJ, Getz G, Hacohen N. Molecular and genetic properties of tumors associated with local immune cytolytic activity. Cell. (2015) 160:48–61. doi: 10.1016/j.cell.2014.12.033

Teschendorff AE, Breeze CE, Zheng SC, et al. A comparison of reference-based algorithms for correcting cell-type heterogeneity in Epigenome-Wide Association Studies. BMC Bioinf. (2017) 18:105. doi: 10.1186/s12859-017-1511-5

Xie T, Solorzano J, Madrid-Mencia M, et al. GEM-DeCan: Improved tumor immune microenvironment profiling through novel gene expression and DNA methylation signatures predicts immunotherapy response. biorxiv. (2023).
